# Phosphorylation of *Drosophila* CENP-A on serine 20 regulates protein turn-over and centromere-specific loading

**DOI:** 10.1093/nar/gkz809

**Published:** 2019-09-19

**Authors:** Anming Huang, Leopold Kremser, Fabian Schuler, Doris Wilflingseder, Herbert Lindner, Stephan Geley, Alexandra Lusser

**Affiliations:** 1 Institute of Molecular Biology, Biocenter, Medical University of Innsbruck, Austria; 2 Institute of Clinical Biochemistry, Biocenter, Medical University of Innsbruck, Austria; 3 Institute of Developmental Immunology, Biocenter, Medical University of Innsbruck, Austria; 4 Institute of Hygiene and Medical Microbiology, Medical University of Innsbruck, Austria; 5 Institute of Pathophysiology, Biocenter, Medical University of Innsbruck, Austria

## Abstract

Centromeres are specialized chromosomal regions epigenetically defined by the presence of the histone H3 variant CENP-A. CENP-A is required for kinetochore formation which is essential for chromosome segregation during mitosis. Spatial restriction of CENP-A to the centromere is tightly controlled. Its overexpression results in ectopic incorporation and the formation of potentially deleterious neocentromeres in yeast, flies and in various human cancers. While the contribution of posttranslational modifications of CENP-A to these processes has been studied in yeast and mammals to some extent, very little is known about *Drosophila melanogaster*. Here, we show that CENP-A is phosphorylated at serine 20 (S20) by casein kinase II and that in mitotic cells, the phosphorylated form is enriched on chromatin. Importantly, our results reveal that S20 phosphorylation regulates the turn-over of prenucleosomal CENP-A by the SCF^Ppa^-proteasome pathway and that phosphorylation promotes removal of CENP-A from ectopic but not from centromeric sites in chromatin. We provide multiple lines of evidence for a crucial role of S20 phosphorylation in controlling restricted incorporation of CENP-A into centromeric chromatin in flies. Modulation of the phosphorylation state of S20 may provide the cells with a means to fine-tune CENP-A levels in order to prevent deleterious loading to extra-centromeric sites.

## INTRODUCTION

CENP-A is a variant of histone H3 that is used by eukaryotic cells for the packaging of centromeric DNA by replacing H3 in centromeric nucleosomes. As such it epigenetically defines the centromere and provides a platform for the assembly of the kinetochore, which in turn is required for microtubule attachment during mitosis and faithful segregation of sister chromatids during cell division (1–6). CENP-A is incorporated into centromeric chromatin in a replication-independent but cell-cycle regulated manner (7). In mammalian cells, CENP-A assembly occurs in early G1 phase following the recruitment of the Mis18 complex during late mitosis by centromeric CENP-C. Mis18 then enables association of the CENP-A chaperone HJURP and deposition of CENP-A (6,8–11). Recent findings also implicated transcription of centromeric DNA as a key mechanism in centromeric chromatin establishment and maintenance (12–16). In *Drosophila melanogaster*, a popular model organism to study centromeric chromatin, CENP-A is incorporated during mitotic metaphase in S2 cells or anaphase in early embryos (17,18).

The composition of centromeres and kinetochores differs considerably between flies and mammals, and several factors that govern centromeric chromatin assembly are not conserved. The fly counterpart of the human CENP-A chaperone HJURP in terms of function although not sequence is CAL1 (19–21). CAL1 binds to pre-nucleosomal CENP-A/H4 dimers, associates with the centromere and mediates CENP-A deposition both *in vitro* and *in vivo* (18,21,22). Besides CAL1, pre-nucleosomal *Drosophila* CENP-A can be detected in complexes with the transcription- and replication-associated histone chaperone FACT, the histone chaperone CAF1 and the histone acetyltransferase HAT1 (23–25). This situation is reminiscent of the processing of canonical H3/H4 dimers prior to chromatin assembly as these too are present in various pre-loading complexes (26). At present it is unclear if, when and how CENP-A is transferred to different pre-loading complexes in the course of deposition. However, the crucial role of CAL1 for CENP-A incorporation into chromatin has been clearly established (19–21). CAL1 also interacts with the inner kinetochore protein CENP-C (22), and CENP-A, CAL1 and CENP-C show mutual dependence with regard to their deposition onto chromatin (20). Similar to mammalian CENP-A assembly, transcription and RNA transcripts are also involved in incorporation and maintenance of fly CENP-A at centromeres (27–32).

In contrast to other histone H3 variants, the primary sequence of CENP-A is poorly conserved and has only ∼50% overall sequence similarity to H3. Similar to H3, CENP-A contains a characteristic histone-fold domain (HFD), which represents the most conserved part of the protein, while the N- and C-terminal regions show the strongest degree of divergence from H3 and are not conserved between CENP-A proteins from different species (33). Canonical H3 and the variant H3.3 are subject to a plethora of posttranslational modifications (PTM) occurring at multiple sites mostly in their N-terminal tails that correlate with and/or regulate diverse processes, such as transcription, post-replicative nucleosome assembly or DNA repair (34). By contrast, only a handful of posttranslational modification sites and types have been detected on CENP-A from different species. In human CENP-A, three phosphorylation sites in the N-terminal region and one in the HFD have been functionally characterized (S7, S16, S18, S68) and another five sites (T21, T23, S27, S32, S41) were detected in global analyses but have not been studied so far (35). The N-terminal glycine residue is subject to trimethylation (36,37), and K124 in the HFD was found to be modified by acetylation, methylation or ubiquitylation in a cell cycle-dependent manner (35,38–41). Several of these modifications have been functionally linked to mitosis, e.g. to regulating chromosome segregation and the assembly of centromere and kinetochore components (G1me3, S7ph, K124ac; (36–39,42–44)), while others have been shown to impact on CENP-A deposition (S18ph; (45)) and interaction with the mammalian CENP-A chaperone HJURP (S68ph, K124ub; (40,46–48)). S16/S18 phosphorylation has been proposed to affect higher order structure of centromeric chromatin (36). However, not all assigned functions are free of dispute (43,49,50) and the clarification of apparent discrepancies is a topic of ongoing research.

In contrast to human CENP-A, much less is known about PTMs in the fly (7). In previous work, we have identified four posttranslational modification sites in the N-terminal region of *Drosophila melanogaster* CENP-A. We found S20, S75 and S77 to be phosphorylated and K105 to be acetylated in pre-nucleosomal CENP-A (23). Notably, K105ac was only found in the cytoplasmic fraction, while S20p and S75p were present in both cytoplasmic and nuclear fractions. S77p, by contrast, was exclusively detected in non-chromatin bound nuclear CENP-A (23). In light of these findings we proposed that differential modification might regulate the deposition pathway of CENP-A in the fly (23). Thus, we initiated studies to elucidate the functions of the identified PTM sites. Here, we examined the impact of S20 phosphorylation on CENP-A function. Analysis of chromatin-bound CENP-A revealed an enrichment of S20p in this fraction. We identified casein kinase II (CKII) as the enzyme responsible for S20 phosphorylation, and we used phosphomimetic and non phosphorylatable mutant CENP-A to examine the effect of S20 phosphorylation on steady-state chromatin levels and assembly dynamics. Most importantly, we demonstrate that S20 phosphorylation is a critical regulator of CENP-A protein stability which contributes to limiting aberrant ectopic incorporation of CENP-A.

## MATERIALS AND METHODS

### Cell lines

*Drosophila* S2 cells were cultured in Schneider's *Drosophila* medium (Gibco) containing 10% FBS (Gibco) and 1% Penicillin/Streptomycin. *Drosophila* S2 cell lines were engineered to contain stably integrated constructs for inducible expression of SNAP (S), Strep-Flag (SF) and GFP-tagged wt and mutant CENP-A fusion proteins. To this end, S2 cells were transfected with expression constructs made with a modified pMT that contains a puromycin resistance cassette (51), and selection of stably integrated constructs was performed by addition of 10 μg/ml puromycin (Sigma) to the culture medium for 2–4 weeks. For further culture of selected cells, the concentration of puromycin was lowered to 2 μg/ml. Overexpression of transgenic CENP-A was induced for 16 h by addition of 0.5 mM CuSO_4_. In all other instances, analyses were performed on cell lines that were not induced but expressed CENP-A at low levels due to leakiness of the MT promoter.

### Plasmid constructs

The generation of constructs for the expression of full length wild-type (wt) CENP-A fused at its N-terminus to a SNAP, Strep-Flag or GFP epitope tag was described previously (23). These constructs were used for PCR-based site-directed mutagenesis to recode serine 20 into alanine (S20A) or aspartate (S20D), respectively. To generate RNAi-resistant CENP-A transgenes, the region between nucleotides 279–678 of the CENP-A coding region was replaced with synthetic DNA molecules containing multiple silent mutations in the constructs harboring epitope-tagged wild-type and S20-mutant sequences.

### Antibody production and validation

Antibodies specifically recognizing phosphorylated S20 in CENP-A were raised in rabbits against a peptide spanning amino acids 15–29 of CENP-A phosphorylated at S20 (ANNSK**pS**PNDDDTAFR) by Davids Biotechnology GmbH (Regensburg, Germany). Final sera were affinity purified against the immunizing peptide and depleted for non-phospho-specific antibodies by purification against the peptide in its non-phosphorylated form (Davids Biotechnology GmbH). Specificity tests were performed by ELISA and by western blotting. Detailed procedure in Supplementary Information.

### Protein extract preparation

To examine the levels of endogenous CENP-A or of induced or non-induced transgenic CENP-A in different cellular compartments, cytoplasmic, nuclear and chromatin extracts were prepared exactly as described before (23) with minor modifications. To examine degradation of total CENP-A, whole cell protein extracts (WCE) were prepared. Total protein extracts from *Drosophila* embryos were prepared by lysing them in 1× SDS loading buffer. Chromatin extracts of FACS-sorted cells were prepared by lysis in RIPA buffer, centrifugation and resuspension of the pellet in 1× SDS loading buffer. Experimental details and buffer compositions are given in Supplementary information.

### Flag-affinity purification of SF-tagged CENP-A

Anti-FLAG M2 Magnetic Beads (M8823; Sigma) were equilibrated in the corresponding extract buffer and incubated with the protein extracts at ∼30 μl beads per 10 ml cytoplasmic or 5 ml nuclear extract overnight at 4°C on a rotating wheel. Subsequently, beads were washed 4 times with 1 ml Wash Buffer (20 mM Tris-HCl pH 7.9, 150 mM NaCl, 2 mM MgCl_2_, 0.2 mM EDTA, 15% glycerol, 0.2 mM PMSF, 1 mM DTT, 10 mM β-glycerophosphate, 0.5 mM benzamidine) followed by elution for 1 h with rotation in 100 μl TBS buffer (30 mM Tris–HCl pH 7.4, 150 mM NaCl, 1 mM EDTA) containing 100 μg/ml Flag peptide (F3290; Sigma).

### Mass spectrometry and data processing for PTM mapping

To identify post-translational modifications of chromatin-bound SF-CENP-A, SF-CENP-A purified by Flag-affinity beads from chromatin extracts was separated by SDS-PAGE, and the CENP-A band was excised. Proteolytic digestion with trypsin and MS analysis were performed as described before (23), with minor modifications: Samples were analyzed by reversed phase C18 HPLC coupled to a Q Exactive HF mass spectrometer (Thermo Scientific). Data analysis was performed using Proteome Discoverer 2.2 (Thermo Scientific) with search engine Sequest.

### Western blotting

**Figure 1.**
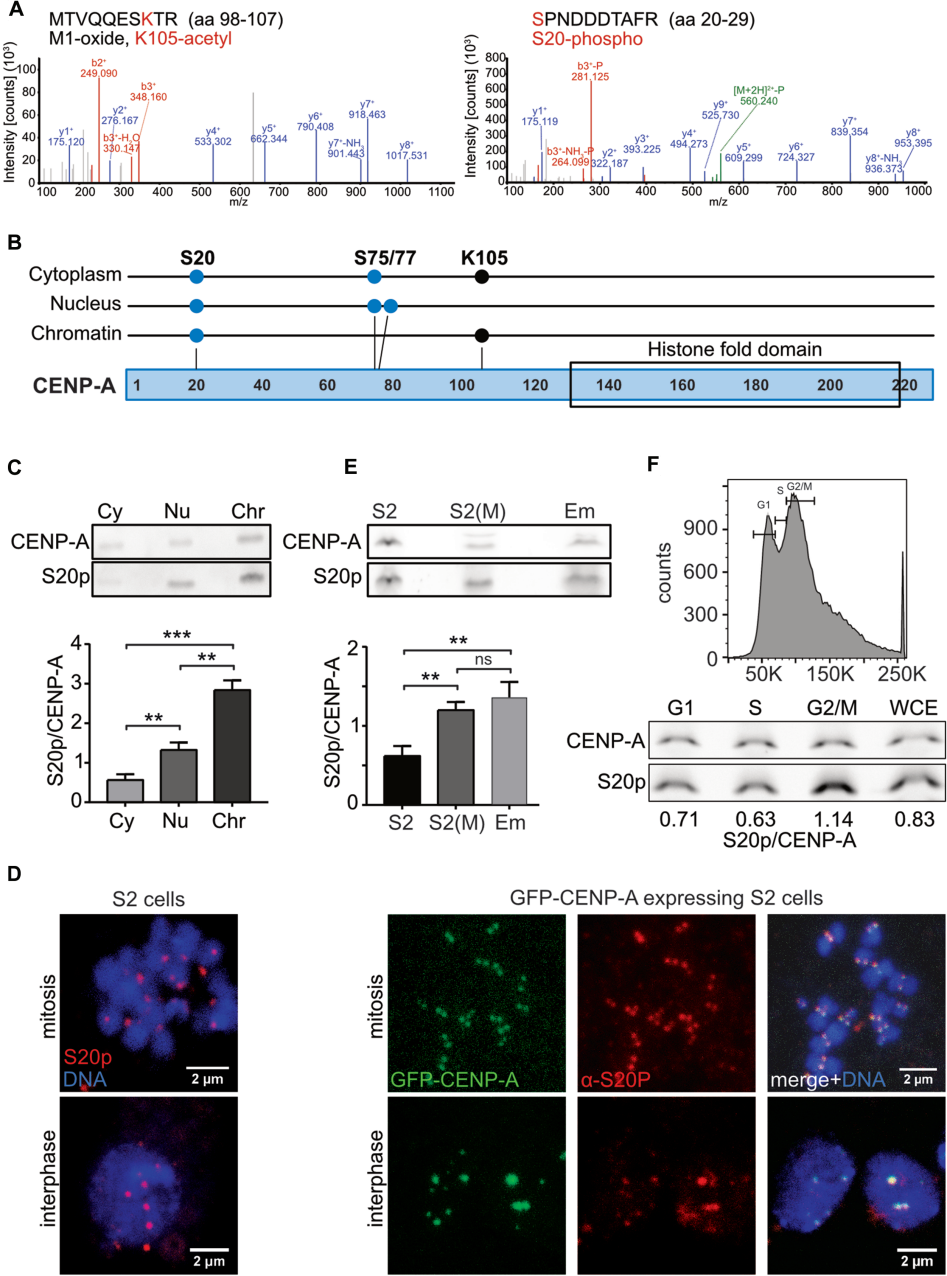
S20 is phosphorylated in soluble as well as chromatin-bound CENP-A. (**A**) MS2 spectra of posttranslationally modified peptides detected in chromatin-bound CENP-A. Peptide sequences and positions within full-length CENP-A are denoted on top. Modified amino acids are shown in red. (**B**) Schematic depiction of posttranslational modification sites on soluble (cytoplasm, nuclear) and chromatin-bound CENP-A. Blue dots symbolize phosphorylation and black dots acetylation. The histone fold domain is indicated. (**C**) *Upper panel*, western blot of cytoplasmic (*Cy*), nuclear (*Nu*) and chromatin (*Chr*) extracts from S2 cells incubated with α-CENP-A or α-S20p antibody. Endogenous CENP-A was immunoprecipitated from cytoplasmic and nuclear extracts and loaded into two wells on the same gel. Chromatin extracts were loaded directly. After blotting, the membrane was cut in half for parallel detection of CENP-A and phospho-CENP-A. *Lower panel*, Quantification of western blot signals. Mean ± SEM ratios of S20p over total CENP-A signal are shown. Unpaired *t*-test was performed to determine statistical significance (*n* = 4; ** *P*< 0.01, *** *P*< 0.001, ns, not significant). (**D**) *Left panels*, Immunofluorescence image of a mitotic (top) and an interphase (bottom) wild-type S2 cell stained with α-S20p antibody. *Right panels*, immunofluorescence images of S2 cells expressing GFP-CENP-A_ wt at endogenous levels. CENP-A was visualized by the GFP signal, phospho-S20 was stained with α-S20p antibody. DNA was detected by DAPI staining. (**E**) Same as in (C) except that whole cell extract from S2 cells, mitotically arrested S2 cells (*S2(M)*) and 0–12 h embryos (*Em*) was analyzed (*n* = 5). (**F**) *Upper panel*, FACS profile of wild-type S2 cells showing the gates (G1, S, G2/M) for cell sorting. *Lower panel*, western blots of chromatin extracts from FACS-sorted cells. Blots were processed as in E (*n* = 1).

Protein samples were separated on 10–12% SDS-PAGE gels and blotted to nitrocellulose membrane. Antibody incubation and detection were performed as described in Supplementary Information. The following antibodies were used: mouse α-Flag (1:5000; Proteintech 20543-1-AP), rabbit α *Drosophila* CENP-A (1:2000; Abcam ab10887), rabbit α-H3 (1:20 000; Millipore 07-690), mouse α-tubulin (1:10 000; Sigma T5168), rabbit α-CENP-A-S20p (1:1000; this study). Secondary anti-rabbit and anti-mouse IgG antibodies (Sigma) coupled to horseradish peroxidase (HRP) were used at 1:10 000. Raw images of western blots from Figures 1E and 4C are shown in Supplementary Figures S10 and S11.

### Immunofluorescence microscopy

S2 cells were settled down on cover slips for 30 min and fixed with 3.7% paraformaldehyde in PBS/0.3% Triton-X100 for 12 min. Cells were blocked in 5% bovine serum albumin (BSA) in PBS, washed and incubated with primary antibody diluted in 5% BSA/PBS, followed by three washing steps and secondary antibody incubation. DNA was stained with 5 μg/ml DAPI (Roche) in PBS followed by three washes in PBS. Cells were mounted in Vectashield and imaged on a Leica TCS SP5 instrument. To analyze mitotic chromosomes of S2 cells, cells were treated with 75 mM KCl before fixation and antibody incubation. The following primary antibodies were used: α-CENP-A (1:500; Abcam ab10887), α-CENP-C (1:500; kind gift from Dr Christian Lehner), α-CENP-A-S20p (1:200, home-made), α-tubulin (1:2000, Sigma T5168). Experimental details are described in Supplementary Information.

### Fluorescence labelling of SNAP-CENP-A

To determine steady-state levels of SNAP-tagged CENP-A wt and mutant proteins at centromeres, cells were incubated with 3 μM SNAP-Cell^®^ TMR Star (NEB) for 30 min. Non-reacted TMR Star was washed out by incubating the cells in fresh medium for 10 min before removing the medium and repeating the washing steps three times. Cells were settled on polylysine-coated coverslips and fixed in 3.7% paraformaldehyde/0.3% Triton-X100 in PBS for 12 min, nuclei were counterstained with 5 μg/ml DAPI and finally mounted in 8 μl Vectashield (Vector). Confocal fluorescence microscopy was performed on a Leica TCS SP5 instrument.

### Proliferation assay

Cells were counted by TC20™ cell counter (Bio-Rad) and diluted to 1 × 10^6^ cells/ml in 2 ml fresh culture medium in six-well plates coupled with gentle shaking. Cell concentrations were measured every 24 h for 3 days. Doubling times were calculated using the exponential growth function of Prism 7.0 (Graphpad), and statistical significance was determined by one-way ANOVA.

### Pulse-chase expression and FRAP experiments

GFP-CENP-A S2 cells were subjected to an expression pulse by addition of 0.5 mM CuSO_4_ for 8 h. Then CuSO_4_ containing medium was removed, cells were washed 3 times for 10 min with fresh medium and left to grow for an additional 16 h (chase). Samples were collected after the expression pulse and after the chase period and processed for confocal fluorescence microscopy. GFP-CENP-A_wt expressing cells after 8 h of induction were used to define a threshold for overexpression that was applied to all images from all samples using ImageJ. The numbers of overexpressing cells relative to total cells (determined by DAPI signal) were calculated and statistical differences between the 8 and 16 h time points were calculated by unpaired t-test (Prism).

For FRAP experiments, GFP-CENP-A expressing S2 cells were either directly used (no overexpression; Figure 8C) or induced by 0.5 mM CuSO_4_ for 16 h. Cells were allowed to attach to coverslips for 10 min, and subsequently overlaid with agarose sheets according to the method by (52). FRAP experiments were performed on a Leica TCS SP5 instrument using a 63×/1.40 oil objective. Photobleaching of the GFP signal was performed by the built-in FRAP-wizard function of the LAS AF software using an argon laser (80% laser power; 10 × 1.293 s/frame). Image stacks of 10–20 z-sections of 0.13 μm were captured before, right after bleaching and at 10, 20 and 30 min after bleaching. Four sections each were merged for analysis. Calculation of signal recovery and further experimental details are described in Supplementary Information.

### Microscopy settings and data analysis

Fixed cells and live cells (FRAP) were imaged on a Leica TCS SP5 confocal fluorescence microscope with 60–85 and 10–20 z stacks, respectively, of 0.13 μm z-sections using a 63x/1.40 oil objective. Mitotic chromosomes were imaged on a Leica TCS SP8 confocal microscope with 30–40 z stacks of 0.13 μM using a 100×/1.40 oil objective. 3D images were reconstructed and analyzed by Imaris v5.1. Using the Arena function of Imaris, one image of the CENP-A wild type group was selected to define cells, nuclei and centromeric spots and those settings were applied to all images of the same experiment to ensure identical image processing. A total of 900–1713 centromeres per experiment were analyzed in this manner. For comparison of the intensity of S-CENP-A in the different cell lines, all spot intensities within one nucleus were summarized and plotted. Mean ± SEM TMR intensities per nucleus were calculated and statistical significance was determined by Mann-Whitney-test using Prism 7.0 (Graphpad) software.

### Protein degradation assay

S2 cells were grown in 50 ml round-bottom flasks containing 15 ml culture medium with gentle stirring. Overexpression of SF-tagged wt and S20-mutant CENP-A was induced by addition of 0.5 mM CuSO_4_ for 16 h. Then, cycloheximide (CHX; Sigma) was added to a final concentration of 40 μg/ml for 4 h to inhibit protein synthesis. Subsequently, aliquots of ∼10^9^ cells were removed from the flask at every 2 h and collected by centrifugation at 1000 × g for 5 min followed by a wash in PBS. WCE was prepared as described in Supplementary Information. For experiments testing the effect of the proteasome on CENP-A degradation, MG132 (Sigma) was added for 5 h to a final concentration of 20 μM; Protein signal intensities were analyzed using ImageJ software, and half-life and statistical calculations were done in Prism 7.0.

### Kinase assay

*In vitro* kinase assays with CKII were performed in a reaction volume of 25 μl for 1 h at 30°C. 1000 units of recombinant CKII (NEB) were incubated in 1× CKII reaction buffer (NEB) with 200 μM ATP, 0.5 μl gamma-^32^P-ATP (10 mCi/ml; Hartmann Analytics GmbH) and 10 μg of peptide p20 (ANNSK**S**PNDDDTAFR) spanning amino acids 15–29 of CENP-A. As negative controls, reactions containing a peptide phosphorylated at S20, p20P (ANNSK**pS**PNDDDTAFR) or reactions without peptide or CKII, respectively, were performed. Reactions were then spotted onto P81 filter paper (Whatman), filters were washed three times for 10 min each in 500 ml 0.75% phosphoric acid, once in 200 ml acetone for 5 min and dried at RT. ^32^P incorporation was measured in a scintillation counter (HITACHI AccuFLEX LSC-8000). In addition, reaction products were analyzed by nano-LC mass spectrometry.

### RNAi experiments

dsRNA probes against 15 different *Drosophila* kinases, Ppa and CENP-A were selected using the algorithm at DRSC/TRiP Functional Genomics Resources (https://fgr.hms.harvard.edu/fly-cell-based-rnai). In addition, nucleotide region 48–573 of the coding sequence of the bacterial Tet repressor (TetR) was chosen as a negative control. Generation of double-stranded RNA probes and RNAi treatment were performed exactly as described previously (23). Knock-down efficiency was evaluated by reverse-transcription quantitative PCR (RT-qPCR) on the last day of treatment. Primer sequences used for the generation of dsRNA templates are available upon request. Experimental details are described in Supplementary Information.

### FACS analysis

To determine cell cycle profiles, 10^5^–10^6^ S2 cells were harvested by centrifugation fixed in ice-cold ethanol abs, collected by centrifugation and resuspended in 1 ml PBS containing 0.1 mg/ml RNase A and 50 μg/ml propidium iodide (PI). To determine the percentage of mitotic cells in wt and mutant cell lines, 5 × 10^5^ cells each were fixed in 1 ml ice-cold 70% ethanol. Cells were washed and incubated with 1% BSA/PBS containing Alexa Fluor^®^ 488-coupled α-Histone H3 Phospho (Ser10) antibody (1:50; Biolegend) at room temperature for 60 min. DNA was stained by addition of 5 μg/ml DAPI. For live cell sorting, 3.2 × 10^6^ cells were stained with 5 μM Hoechst prior to sorting according to their DNA content. Flow cytometric analysis or cell sorting of single cell suspensions of S2 cells were performed on an LSR Fortessa or a FACS-Aria-III, respectively, (both BD) instrument and analyzed using FlowJo^®^ v10 software.

## RESULTS

### S20-phosphorylated CENP-A is enriched in chromatin

In our previous work, we identified three phosphorylation sites (S20p, S75p, S77p) and one acetylation site (K105ac) in CENP-A preparations from cytoplasmic and nuclear extracts of *Drosophila* S2 cells (23), yet the modification state of chromatin-bound CENP-A remained unknown. We therefore purified CENP-A from chromatin extracts of a cell line stably expressing Strep-Flag-tagged CENP-A (SF-CENP-A) (Supplementary Figure S1A) for analysis by mass-spectrometry. We found that chromatin-associated SF-CENP-A was not modified at S75/77 but showed phosphorylation at S20 and acetylation at K105 (Figure 1A). Because S20 phosphorylation was the only modification detectable in soluble (23) as well as in chromatin-bound fractions of CENP-A (Figure 1B), we focused our further studies on this modification site. To examine the S20 phosphorylation status of endogenous CENP-A, we raised an antibody against a phosphorylated peptide spanning S20 (Supplementary Figure S1B) and performed western blot analysis of cytoplasmic, nuclear and chromatin fractions of wild-type S2 cells. The results revealed that the portion of phosphorylated S20 relative to total CENP-A was highest in chromatin-bound CENP-A (Figure 1C). Likewise, immunostaining with the α-S20p antibody showed clear centromeric signals in interphase and mitotic cells in both wild-type S2 cells and cells expressing GFP-tagged CENP-A (Figure 1D). When we arrested S2 cells in mitosis by colchicine treatment (Supplementary Figure S1C) the relative amount of chromatin-bound S20-phosphorylated CENP-A increased (Figure 1E). These results were confirmed by western blot analysis of cells sorted according to their cell cycle stage (G1, S and G2/M; Figure 1F) suggesting that S20 phosphorylation might be important during mitosis and/or for the loading of CENP-A which takes place during mitosis (18). Consistent with this idea, the ratio between phosphorylated and total CENP-A in *Drosophila* embryos, where most cells are cycling cells/nuclei therefore requiring continuous CENP-A loading, was similar to that of mitotically arrested S2 cells (Figure 1E, F).

### Casein kinase II phosphorylates CENP-A at S20 *in vivo* and *in vitro*

**Figure 2.**
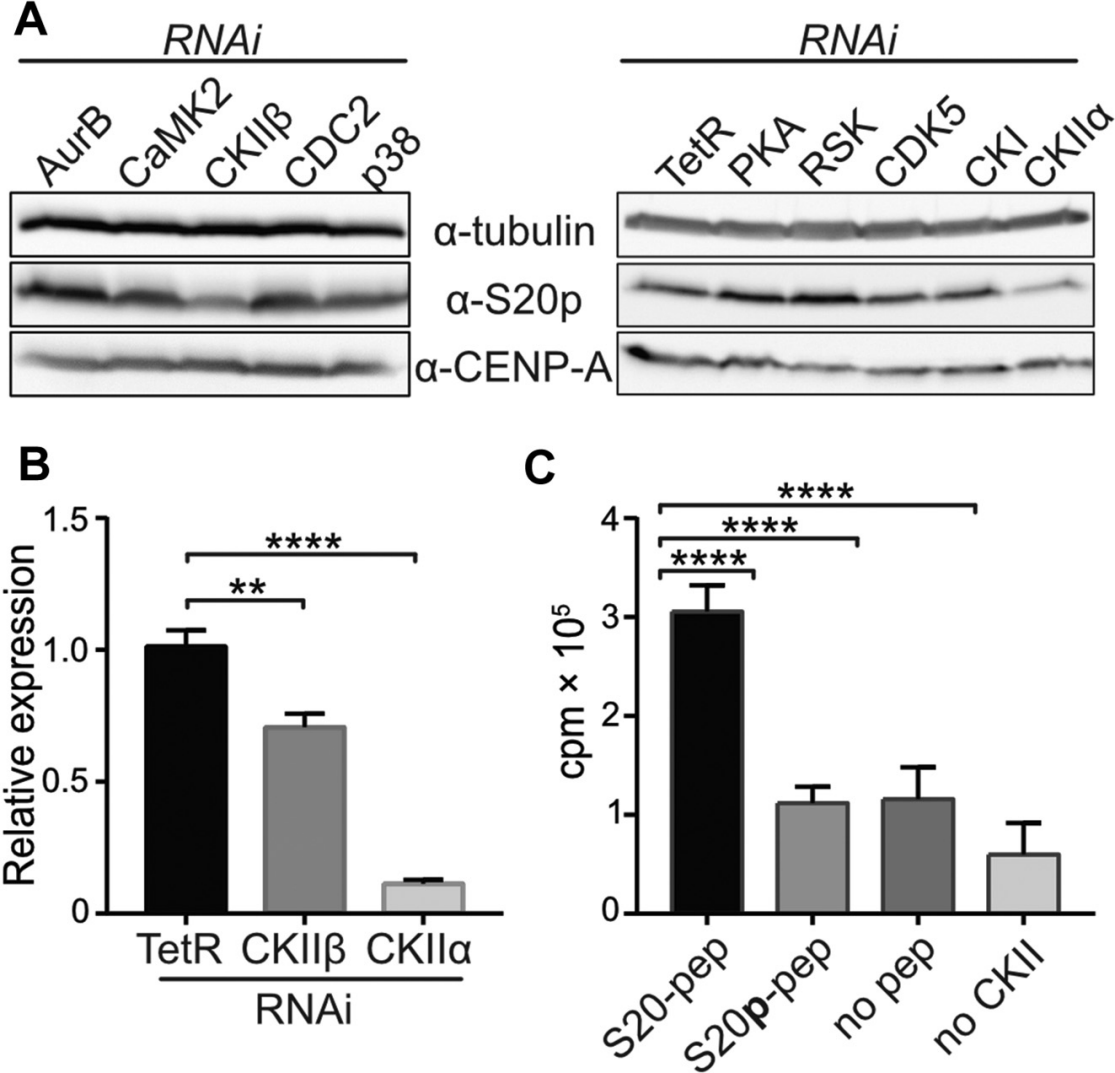
CKII phosphorylates CENP-A at S20. (**A**) RNAi against CKIIα and CKIIβ results in a decrease of α-S20p signal on western blots of S2 whole cell extracts. Cells were treated with dsRNA against the indicated kinases or control TetR for 4 days before whole cell extract (WCE) preparation and western blotting. Samples were loaded in replicates to two gels. One membrane was detected with α-S20p and the other with α-CENP-A. α-tubulin was used to control for equal loading on both membranes but only one data set is shown. (**B**) Reverse-transcription real-time PCR analysis of knock-down efficiency of CKII subunit transcripts in cells treated with the respective dsRNAs or with control TetR RNA. (**C**) Kinase assay with recombinant CKII and CENP-A peptides spanning S20. ^32^P-incorporation was measured by liquid scintillation counting. Reactions containing phosphorylated S20 peptide (S20p), no peptide or no CKII were performed as negative controls. Note that the increase in ^32^P-incorporation observed in negative control reactions containing CKII is due to the known autophosphorylation activity of CKII. Unpaired *t*-test was performed to determine statistical significance of differences (**P< 0.01, ****P< 0.0001).

We next asked which kinase mediates CENP-A-S20 phosphorylation. To this end, we carried out phosphorylation motif analysis using NetPhos3.1 (53). Significant matches with recognition motives of 15 serine/threonine kinases were obtained. We then performed an RNAi screen targeting each of these kinases in S2 cells and monitored reduction of CENP-A-S20 phosphorylation by immunoblotting of whole cell extracts. The knockdown of nine of these kinases by RNAi was successful and resulted in >80% reduction in their transcript levels (Supplementary Figure S2A) but only the knock-down of both subunits of casein kinase II (CKII) caused a strong decrease of S20 phosphorylation (Figure 2A). The reduction of S20p signal was stronger in CKIIα knockdown cells, most likely due to the more efficient depletion of CKIIα compared to CKIIβ, which was only ∼30% depleted (Figure 2B). These findings were further corroborated by an *in vitro* kinase assay using recombinant CKII and a peptide spanning S20 of CENP-A. CKII incorporated significant amounts of radiolabeled phosphate into the S20 peptide, whereas a pre-phosphorylated S20 peptide was not modified (Figure 2C). Moreover, mass spectrometry analysis of reaction products showed that S20 was one of the major amino acid targets of CKII in the *in vitro* assay. Another product was T26p (Supplementary Figure S2B). However, this is likely due to relaxed specificity of CKII in the *in vitro* assay with a peptide substrate as we never found T26 to be phosphorylated in CENP-A *in vivo*. Of note, we have found CKIIα and β subunits to specifically co-purify with CENP-A in previous tag-affinity purification experiments from S2 cells (data not shown; (23)). Together, the results strongly suggest that CKII is the major kinase for S20 phosphorylation of CENP-A in *Drosophila* S2 cells.

### S20 phosphorylation affects centromeric localization of CENP-A

**Figure 3.**
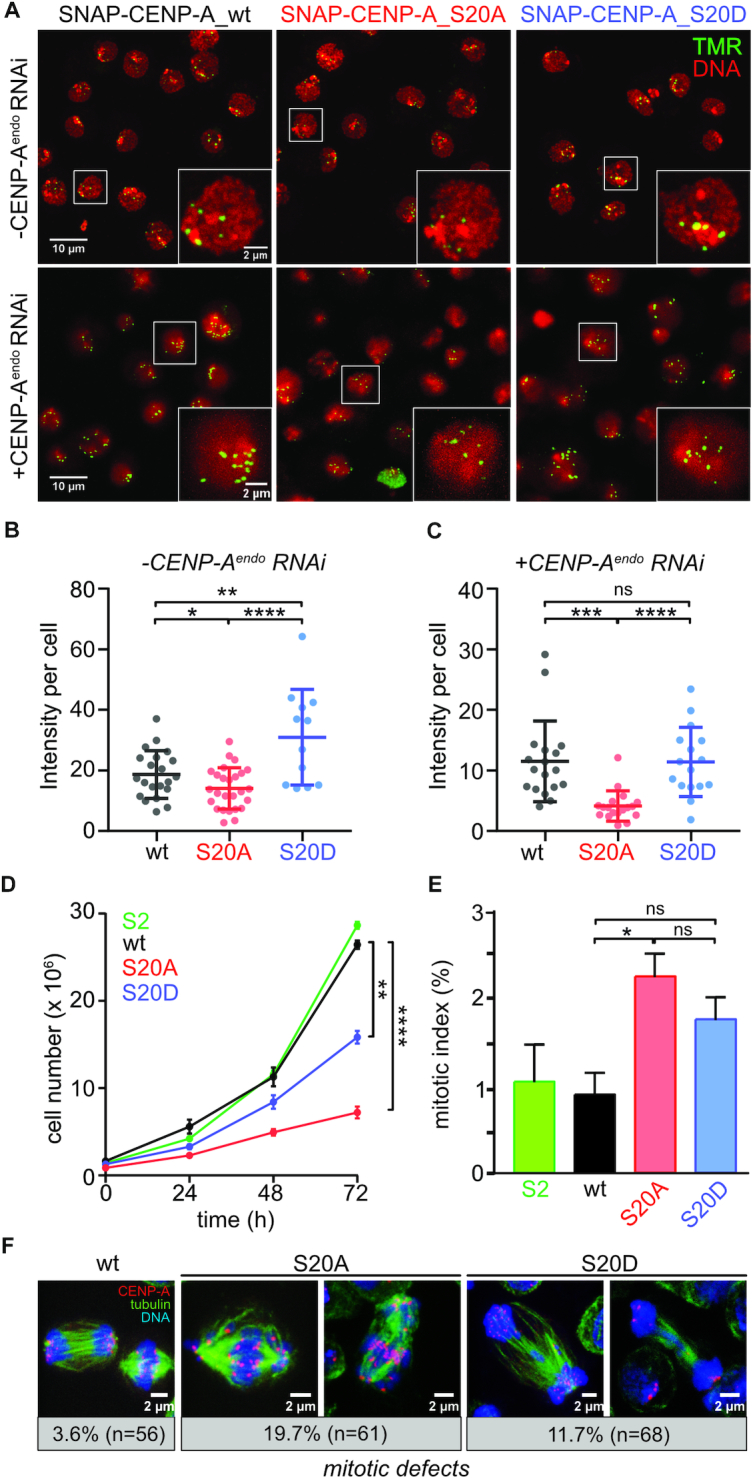
Mimicking the phosphorylated or non-phosphorylated state of S20 interferes with steady-state levels of CENP-A at the centromere. (**A**) TMR-staining of S2 cells expressing low levels of SNAP-tagged wt, S20A and S20D CENP-A in the absence (*top panels*) or presence (*bottom panels*) of RNAi against endogenous CENP-A (CENP-A^endo^). Insets are 3× enlargement of the indicated nuclei. (**B**) Quantification of centromeric TMR signal intensities from (**A**) using Imaris v5.1. software. Three to five images were analyzed per experiment. Statistical significance of differences in cumulative centromere intensities per cell was determined by unpaired *t*-test with a significance threshold of *P*<0.05. Means ± SEM of one representative out of 3 experiments are shown. (**C**) Same as in (**B**) except that cells were treated with dsRNA against endogenous CENP-A. (**D**) Proliferation assay of non-transfected S2 cells (S2 wt) and cells stably expressing SNAP-tagged CENP-A_wt or S20A- and S20D-mutant proteins. Means ± SEM of three biological replicates are shown and statistical difference of doubling times was calculated by one-way ANOVA. (**E**) Mitotic index of cell lines described in (D). Mitotic cells were detected by FACS of α-phospho-H3S10 stained cells. Mean ± SEM percentage of stained relative to total cells is shown. *P< 0.05, **P< 0.01,***P< 0.001, ****P< 0.0001, ns, not significant. (**F**) Imunofluorescence images of examples for mitotic defects in SNAP-CENP-A_wt, S20A and S20D expressing cells (no CuSO_4_ induction). Cells were stained with antibodies against CENP-A and tubulin. DNA was visualized by DAPI. For wt, a normal mitosis is shown. Numbers below the images state the percentage of defective mitoses and the numbers of analyzed mitoses.

To study the functional role of CENP-A phosphorylation at S20, we generated cell lines expressing SF-CENP-A or SNAP-tagged CENP-A (S-CENP-A) in which S20 was mutated to alanine (S20A) to block phosphorylation or to aspartate (S20D) to mimic the phosphorylated state (Supplementary Figure S3A). All transgenes were under the control of a CuSO_4_-inducible metallothioneine promoter. In the absence of CuSO_4_ induction, transgenic CENP-A was expressed at near physiological levels, restricting its incorporation to centromeres as demonstrated by colocalization with the centromeric protein CENP-C (Supplementary Figure S3B). We first examined if steady state centromeric levels of wild-type and mutant versions of S-CENP-A showed differences. Staining of cells with 6-carboxytetramethylrhodamine (TMR Star) and quantification of the resulting centromeric signal intensities per cell revealed a 24.5% reduction of centromeric localization of the non-phosphorylatable mutant S-CENP-A_S20A compared to S-CENP-A_wt. By contrast, signal intensity of phosphomimetic S-CENP-A_S20D was significantly increased (65.9%; Figure 3A, B). Because in our stable cell lines, endogenous CENP-A is present in addition to the transgenic variants, it was possible that centromeric targeting and/or maintenance of CENP-A mutant proteins was aided by the presence of endogenous CENP-A. In fact, it was shown for human cells that the CENP-A chaperone HJURP dimerizes and thereby facilitates the loading of two CENP-A/H4 heterodimers and formation of a nucleosome (54). Although no similar evidence has been reported for *Drosophila* CAL1, we attempted to minimize a potential effect of endogenous CENP-A by RNAi-mediated knock-down. To this end, we modified the nucleotide sequences of CENP-A_wt, CENP-A_S20A and CENP-A_S20D constructs to render them resistant to RNAi against endogenous CENP-A. dsRNA treatment of stable cell lines resulted in ∼40–50% reduction of endogenous CENP-A (Supplementary Figure S3C) but did not reduce transgenic CENP-A expression (Supplementary Figure S3D) and strongly decreased centromeric CENP-A signal in non-transfected S2 cells (Supplementary Figure S3E). Measuring steady state TMR signal intensities in RNAi-treated transgenic cell lines showed a more pronounced decrease (64%) of centromeric signal of the non-phosphorylatable S20A mutant protein compared to S-CENP-A_wt, while no intensity difference was observed for S-CENP-A_S20D (Figure 3A, C). These results suggest that S20 phosphorylation may be required for loading and/or maintenance of CENP-A at the centromere.

Proliferation measurements of the three transgenic lines in the absence of CuSO_4_ induction revealed that S20A and to a lesser extent S20D-expressing cell lines showed reduced proliferation rates compared to S-CENP-A_wt expressing cells (Figure 3D). Consistent with this they exhibited a moderately increased mitotic index although statistical significance was only reached for S20A (Figure 3E). When we examined mitoses more closely, we found that the number of defective mitoses was increased in S20A and S20D expressing cells with S20A causing mostly chromosome congression defects and premature segregation presumably due to cohesion fatigue, while S20D cells more frequently showed lagging chromosomes during anaphase/telophase (Figure 3F). Hence, S20D cells might more often be able to complete mitoses, while S20A cells might tend to experience delay or arrest in mitosis, thus accounting for the different extent in proliferation rate reduction for S20D and S20A expressing cells.

**Figure 4.**
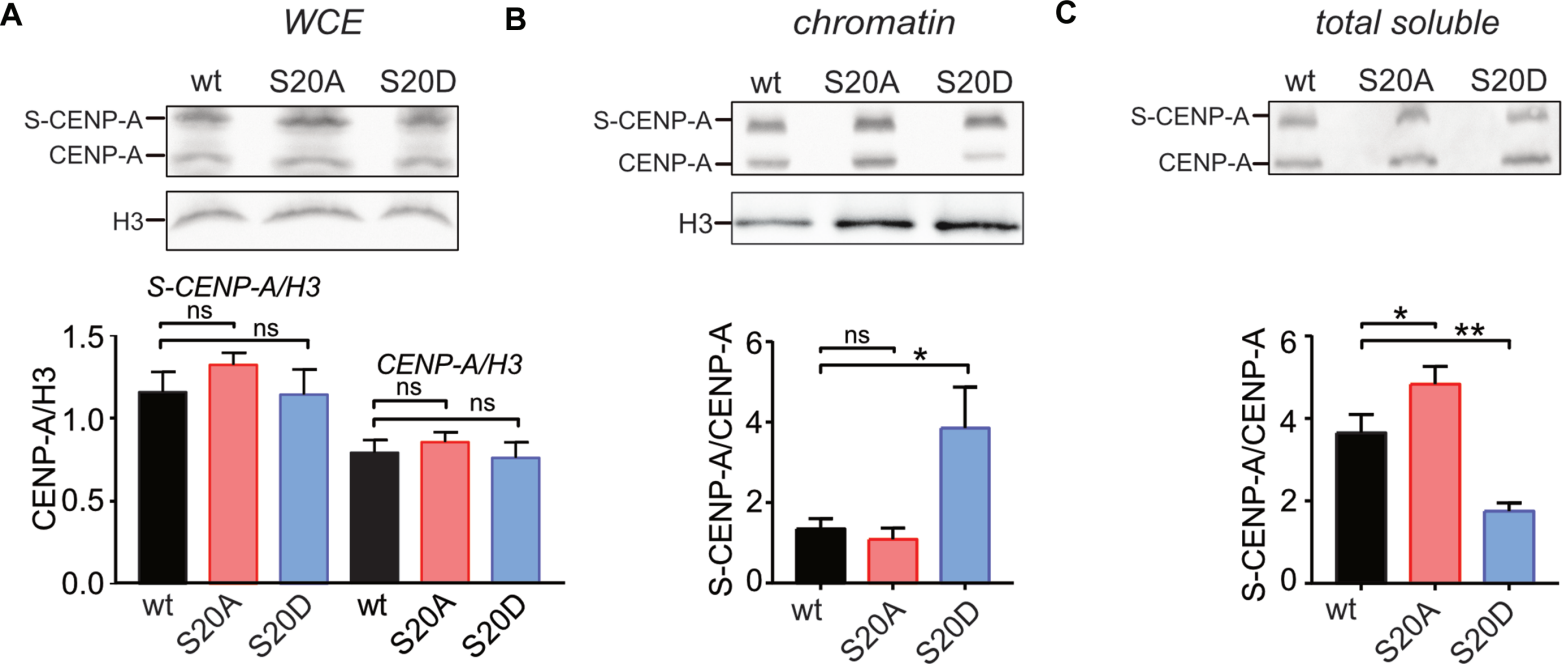
Phosphomimetic CENP-A_S20D is enriched in chromatin but decreased in the soluble fraction. (**A**) *Upper panel:* Representative western blot of whole cell extract (WCE) from cells expressing SNAP-tagged wt, S20A or S20D CENP-A at low levels. α-CENP-A antibody detects transgenic and endogenous CENP-A. α-H3 antibody was used to control for equal loading. *Lower panel:* Quantification of SNAP-CENP-A (S-CENP-A) and endogenous CENP-A western blot signals. Values were normalized to H3 and are shown as mean ± SEM of three independent experiments. (**B**) *Upper panel*: Representative western blot of chromatin extracts incubated with α-CENP-A and α-H3 antibody. *Lower panel*: Quantification of S-CENP-A and endogenous CENP-A signals. Ratios of S-CENP-A/endogenous CENP-A signals are shown as mean ± SEM of three independent experiments. (**C**) Same as in (B) except that total soluble protein was analyzed and that the soluble fraction was concentrated by vacuum evaporation prior to loading. For all panels statistical significance was determined by unpaired *t*-test with a significance threshold of *P*< 0.05. ns, not significant, *P< 0.05, **P< 0.01.

One explanation for the observed differences in centromeric deposition/abundance of wild-type and mutant proteins might be that the transgenes are expressed at different levels. However, when we analyzed whole cell extracts of the three cell lines, we detected similar amounts of protein (Figure 4A). Likewise, the amount of endogenous CENP-A was similar in all three lines (Figure 4A). We also fractionated cells into chromatin and total soluble protein to examine potential differences in distribution. These experiments revealed a striking enrichment of S-CENP-A_S20D in the chromatin fraction when exogenous CENP-A was normalized to endogenous CENP-A (Figure 4B) confirming the results obtained with endogenous CENP-A showing enrichment of S20p in centromeric chromatin (Figure 1C). Interestingly, the soluble portion of S-CENP-A_S20D was significantly smaller than that of S-CENP-A_wt, while that of S-CENP-A_S20A was larger (Figure 4C). To rule out that the SNAP-epitope tag contributed to these effects, we performed the same experiments with cell lines expressing SF-tagged CENP-A versions obtaining very similar results (Supplementary Figure S4). Small differences were observed for the SF-CENP-A_S20A expressing cell line, in which the amount of endogenous CENP-A was increased compared to SF-CENP-A_wt expressing cells (Supplementary Figure S4A).

Taken together, the data suggest two distinct but not mutually exclusive scenarios for the role of S20 phosphorylation: (i) S20p is involved in CENP-A loading and maintenance at the centromere. This idea is supported by the enrichment in chromatin of phosphorylated CENP-A and of the phosphomimetic S20D mutant protein but depletion from chromatin of the non-phosphorylatable CENP-A_S20A. (ii) S20p is required for the degradation of soluble CENP-A. Reduced amounts of phosphomimetic CENP-A_S20D but increased levels of CENP-A_S20A in the soluble protein pool point towards this possibility. To determine which of these possibilities holds true, we examined the potential of S20p to affect CENP-A turn-over.

### Phosphorylation of S20 promotes CENP-A protein degradation

**Figure 5.**
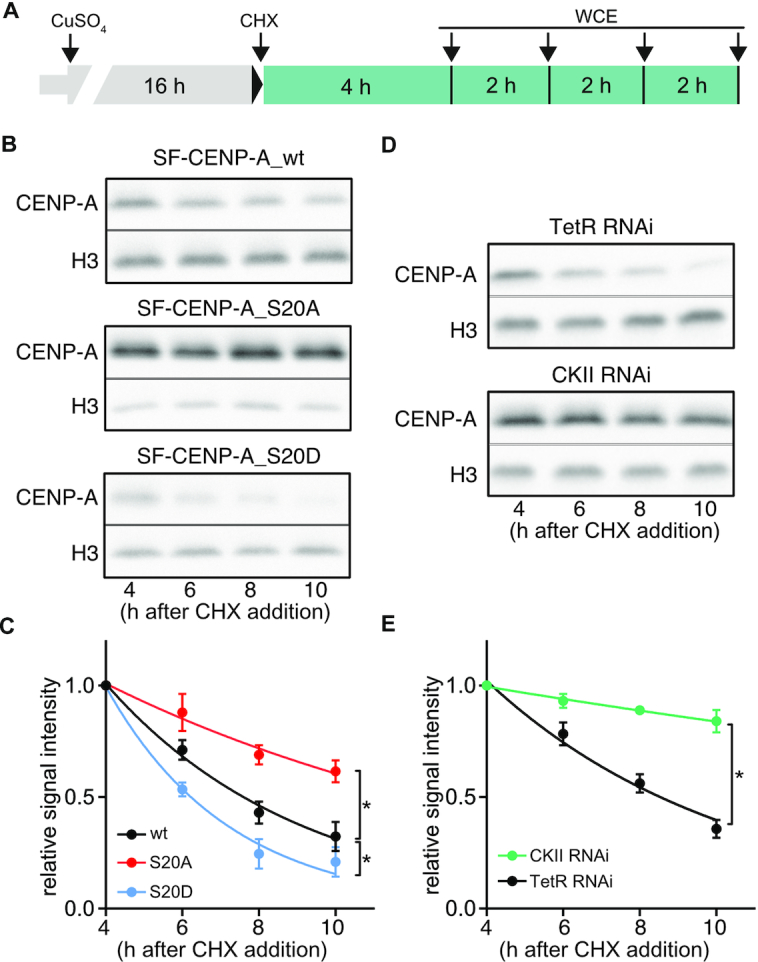
Phosphorylation of S20 promotes degradation of CENP-A. (**A**) Schematic depiction of experimental set-up to examine CENP-A degradation. (**B**) Representative western blots of whole cell extracts (WCE) prepared from S2 cells overexpressing the indicated proteins. Samples were taken at the time points shown in (A). After western blotting, membranes were cut and the upper half was incubated with α-Flag antibodies to detect SF-CENP-A, while the lower was probed with α-H3 as an internal reference. (**C**) Quantification of SF-CENP-A signal intensities. Values of each lane were normalized to the corresponding H3 signal. The resulting value of 4 h after CHX addition was set as 1 and all other time points are shown relative to it to facilitate comparison. Means ± SEM of three independent experiments are shown. To determine statistical significance, protein half-life times were calculated using Prism v7 and values were analyzed by unpaired *t*-test (*n* = 3 independent experiments). Statistical significance (*P*< 0.5) is denoted by asterisks next to the degradation curves. (**D**) Representative western blots of WCE of cells expressing SF-CENP-A_wt and treated with dsRNA against TetR (*top*) or CKII (*bottom*) prepared at the indicated timepoints. Membranes were probed with α-Flag and α-H3 antibodies as described under (B). (**E**) Quantification of SF-CENP-A signal intensities as described under (**C**). Means ± SEM of three independent experiments are shown. *P< 0.05.

CENP-A expression levels must be tightly controlled in the cell, because overexpression of CENP-A leads to its incorporation at ectopic sites and to the formation of neocentromeres and genome instability in yeast and flies (32,55,56). Likewise, overexpression and ectopic localization of CENP-A is a characteristic feature of many types of cancers (32). Proteolysis is known to play an important role in controlling CENP-A levels and restricting CENP-A incorporation to the centromere (32). To investigate the possibility that S20 phosphorylation controls the turn-over of CENP-A, we performed time course experiments to monitor protein stability. To facilitate protein detection, expression of SF-tagged wt and mutant CENP-A was induced for 16 h with CuSO_4_ followed by treatment of cells with cycloheximide (CHX) to inhibit new protein synthesis, and analysis of CENP-A protein abundance in whole cell extracts by western blot with α-Flag antibodies at different time points after addition of CHX (Figure 5A). Histone H3 was used as a loading control. The results showed that the non-phosphorylatable SF-CENP-A_S20A mutant protein exhibited substantially slower degradation kinetics than SF-CENP-A_wt. By contrast, mimicking phosphorylation (S20D) caused significant protein destabilization, indicating that S20 phosphorylation sensitizes CENP-A to degradation (Figure 5B, C). To corroborate these data, we examined CENP-A stability in cells depleted of CKII (Supplementary Figure S5A) to reduce S20p in SF-CENP-A_wt and found remarkable stabilization of the soluble protein compared to control cells treated with dsRNA against the non-expressed bacterial protein TetR (Figure 5D, E). It should be noted, however, that since CKII is a multifunctional enzyme with numerous substrates in the cell (57), we cannot completely rule out indirect effects of CKII knock-down on CENP-A stability (58). However, taken together, our data link the phosphorylation of S20 in CENP-A to its degradation.

**Figure 6.**
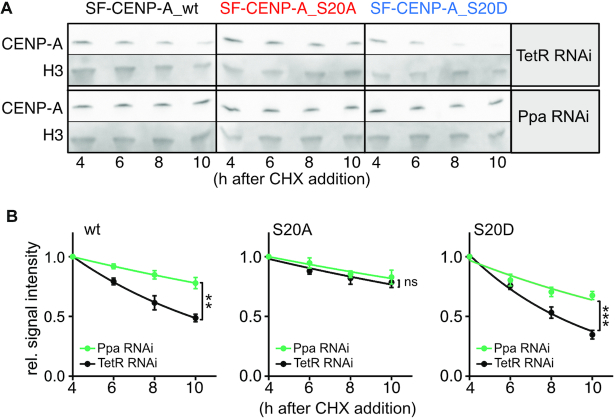
S20 phosphorylation regulates SCF^Ppa^-mediated CENP-A degradation. (**A**) Representative western blots (*n* = 3 independent experiments) of WCE from cells overexpressing SF-CENP-A_wt or S20 mutant proteins prepared at the indicated timepoints (set-up as depicted in Figure 5A). Cells were subjected to RNAi against TetR (control) or Ppa. Membranes were probed with α-Flag antibodies to detect SF-CENP-A, and with α-H3 as an internal reference. (**B**) Quantification of SF-CENP-A signal intensities. Values of each lane were normalized to the corresponding H3 signal. The resulting value of 4 h after CHX addition was set as 1 and all other time points are shown relative to it to facilitate comparison. Means ± SEM of three independent experiments are shown. To determine statistical significance, protein half-life times were calculated and results were analyzed by unpaired *t*-test. Statistical significance. **P< 0.01, ***P< 0.001, ns, not significant.

Proteolysis of *Drosophila* CENP-A was previously reported to require the action of Partner of Paired (Ppa), one of multiple adaptor subunits of E3-ubiquitin ligase SCF. Ppa was shown to interact with *Drosophila* CENP-A, and CENP-A gained increased stability when Ppa was knocked-down by RNAi (59). Because SCF degrons are known to be regulated by phosphorylation (60,61), we examined if S20 phosphorylation regulates SCF^Ppa^ -mediated CENP-A degradation. Knock-down of Ppa by RNAi in S2 cells expressing wt or S20-mutant CENP-A resulted in increased stability of wt CENP-A as reported before (Figure 6A, B; Supplementary Figure S5B; (59)). Importantly, the half-life of phosphomimetic SF-CENP-A_S20D was significantly increased in Ppa knock-down compared to control cells (Ppa RNAi *t*_1/2_ = 6.1 h versus TetR RNAi; *t*_1/2_ = 3.2 h), while SF-CENP-A_S20A half-life was largely unaffected (Ppa RNAi *t*_1/2_ = 13.7 h versus TetR RNAi *t*_1/2_ = 12.4 h, Figure 6A, B). These results strongly suggest that S20 phosphorylation regulates the ability of SCF^Ppa^ to mark CENP-A for degradation.

Altogether, our data demonstrate that the phosphorylation of S20 by CKII sensitizes CENP-A for degradation in a manner that is dependent on the ubiquitin ligase SCF^Ppa^.

### S20 phosphorylation promotes ectopic CENP-A degradation upon overexpression

**Figure 7.**
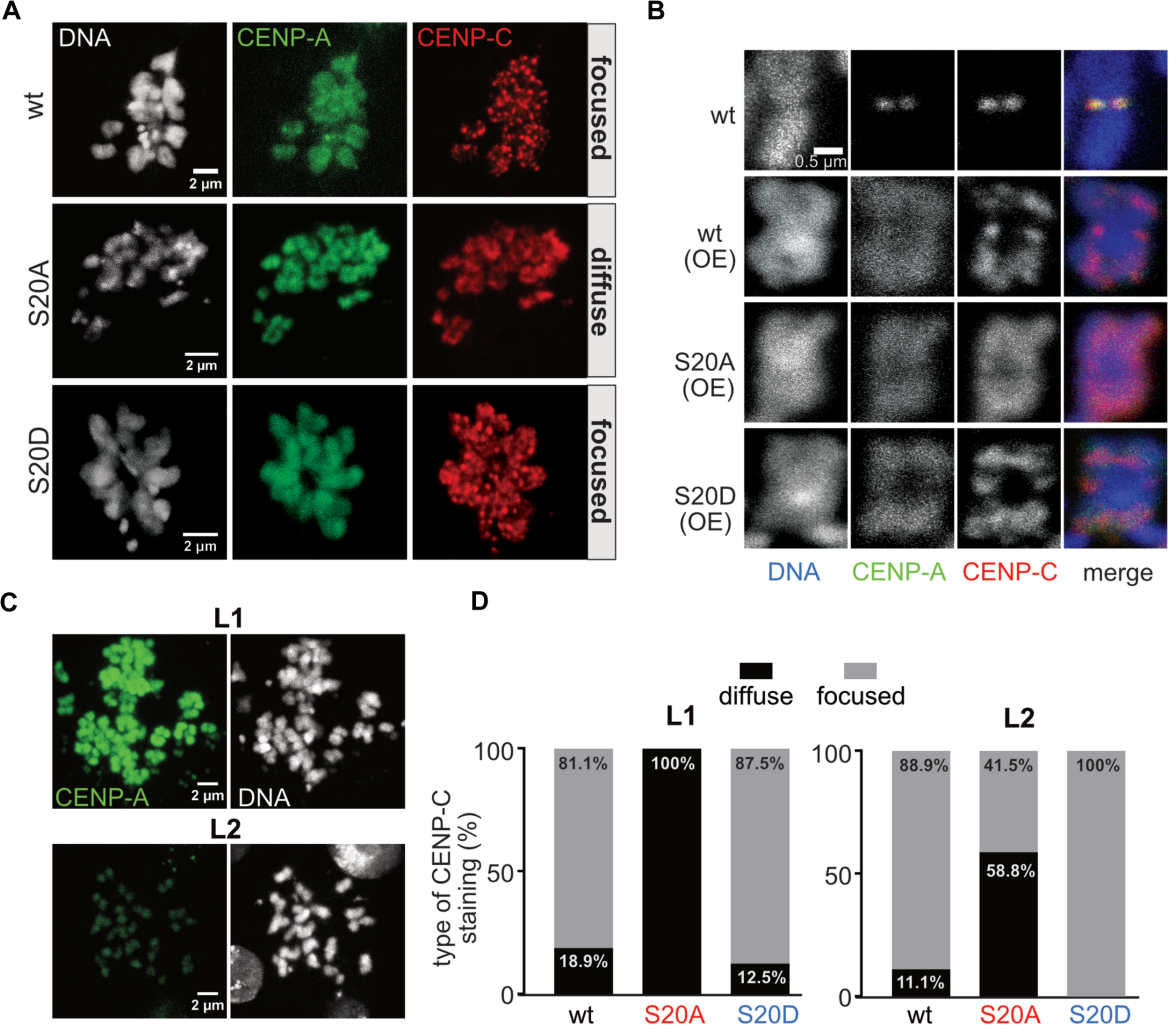
Overexpression of CENP-A mutant proteins results in different types of non-centromeric CENP-C incorporation. (**A**) Example images of mitotic chromosome spreads showing diffuse or focused staining of α-CENP-C upon CENP-A overexpression. CENP-A was visualized by its GFP signal, DNA was stained with DAPI. Images were taken with the indicated cell lines. (**B**) Magnification of individual chromosomes showing focused CENP-C signals along the chromosome arms upon overexpression of wt or S20D proteins but diffuse staining in S20A overexpressing cells. *Top panels* show an image from a cell expressing GFP-CENP-A_wt at endogenous levels. (**C**) Example images of mitotic chromosome spreads upon CENP-A overexpression showing strong (L1) and weak (L2) ectopic incorporation signals. Because of the strong differences in overexpression levels of CENP-A, mitotic chromosome spreads where manually assigned to the L1 and L2 groups using microscopic images generated with identical microscopic settings. The presented images are from GFP-CENP-A_wt expressing cells. CENP-A was detected by its GFP signal, DNA was stained with DAPI. (**D**) Quantification of diffuse and focused CENP-C signals in mitotic chromosomes expressing GFP-CENP-A at L1 (*left*; wt *n* = 53, S20A *n* = 84, S20D *n* = 80) or L2 (*right*; wt *n* = 18, S20A *n* = 17, S20D *n* = 94) level. Assignment of chromosomes to either group was performed as described under C.

It is well established that upon overexpression, CENP-A can be incorporated at ectopic sites which may lead to the formation of neocentromeres (55,56,62,63). To examine a potential effect of S20 phosphorylation on non-centromeric chromatin incorporation of CENP-A, we generated S2 cell lines stably transfected with inducible CENP-A wt and S20 mutant constructs fused to a GFP tag. Similar to the other transgenes used in this study, wt and mutant GFP-CENP-A were expressed at low levels in the absence of induction resulting in their incorporation into centromeres only (Supplementary Figure S6A). Upon overnight induction of GFP-CENP-A overexpression, however, all three fusion proteins displayed diffuse nuclear staining (Supplementary Figure S6B) that remained unaffected by extraction of soluble protein prior to fixation (Supplementary Figure S6C), thus indicating promiscuous incorporation throughout chromatin. To better characterize ectopic incorporation, we co-stained the samples with an antibody against CENP-C and examined CENP-A and CENP-C signals on spreads of mitotic chromosomes. Interestingly, while overexpression of GFP-CENP-A_S20A caused diffuse staining of CENP-C throughout chromatin, GFP-CENP-A_wt and GFP-CENP-A_S20D overexpression resulted in the formation of distinct CENP-C foci in addition to the diffuse background staining (Figure 7A). Moreover, staining of GFP-CENP-A_wt mitotic chromosome spreads with the α-S20p antibody also revealed chromosomes with focused (87%) and others with diffuse staining (13%, n = 47; Supplementary Figure S7). Imaging of individual mitotic chromosomes at higher magnification confirmed the wt- and S20D-specific accumulation of CENP-C at several locations along the chromosome arms, although the CENP-A signal was evenly distributed. In S20A expressing cells, by contrast, CENP-C was found all over the chromosomes in a diffuse pattern matching that of CENP-A (Figure 7B). Because each mitotic chromosome should only have two CENP-C spots corresponding to the centromeres of the sister chromatids (as shown in the top panel of Figure 7B in cells not overexpressing CENP-A), the additional focal CENP-C assemblies observed upon overexpression of S20D and wt CENP-A must be due to non-centromeric incorporation, yet need not necessarily be neocentromeres. For better quantification of this phenotype, we defined two groups of mitotic nuclei with respect to the intensity of their GFP-CENP-A expression levels (L1, strong signal; L2, weak signal; examples for both expression levels are shown in Figure 7C). Strikingly, in cells overexpressing phosphomimetic S20D CENP-A, 88% of mitotic nuclei with L1 intensity and 100% of nuclei with L2 intensity exhibited CENP-C foci. By contrast, in cells overexpressing non-phosphorylatable S20A, foci were detected in only 41% of L2 mitotic spreads and never in L1 chromosomes. GFP-CENP-A_wt overexpressing cells behaved similar to the S20D mutant, although the numbers of mitotic nuclei with CENP-C dot formation were slightly lower (Figure 7D).

**Figure 8.**
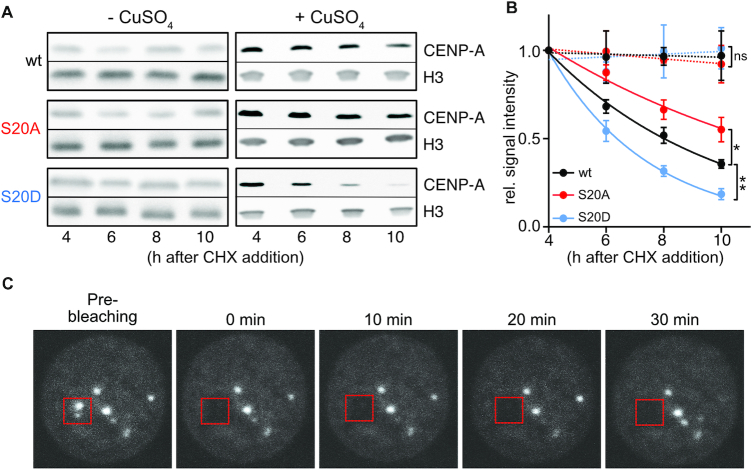
Increased lability of chromatin bound CENP-A_S20D in overexpressing but not in non-overexpressing cells. (**A**) Degradation assay as depicted in Figure 5A using chromatin extracts from cells without or with CuSO_4_ induction. (**B**) Quantification of signals from (A) and statistical evaluation was performed as described for Figure 5C. Dashed lines indicate samples from non-induced cells, solid lines those from induced cells. *P< 0.05, ** *P*< 0.01, ns, not significant. (**C**) FRAP analysis of centromeric GFP-CENP-A_wt signals in non-induced cells. After bleaching, no signal recovery was detected during the observation period.

Based on these findings we speculated that the ability of the phosphomimetic CENP-A_S20D to recruit increased amounts of CENP-C to distinct locations might be determined by its increased turn-over at extra-centromeric locations. In an overexpression situation, this may prevent the general dilution of CENP-C to non-centromeric sites which results in diffuse staining of CENP-C. Instead, S20D (and phosphorylated wt CENP-A) might be cleared more efficiently from certain sites than from others, which would allow the accumulation of CENP-C on the latter and result in the observed focal staining of CENP-C. To test this hypothesis, we first analyzed the stability of centromeric CENP-A by performing Western blot-based degradation assays of chromatin-bound CENP-A in the absence of CuSO_4_ induction. In this way, CENP-A located at centromeres should constitute the predominant form (64,65). The experiments showed that centromeric CENP-A was very stable regardless of whether S20 was mutated to alanine or serine or not mutated at all, since its amount was unaltered during the examined time period (Figure 8A, B). We also used fluorescence recovery after photobleaching (FRAP) to monitor the stability of CENP-A at centromeric locations in cells not overexpressing CENP-A. As expected, no recovery was detected within the measured time period (Figure 8C). In contrast to that, overexpressed CENP-A which is incorporated all over the chromatin, was degraded with kinetics similar to those observed for whole cell extracts (Figure 8A, B compare to Figure 5C).

**Figure 9.**
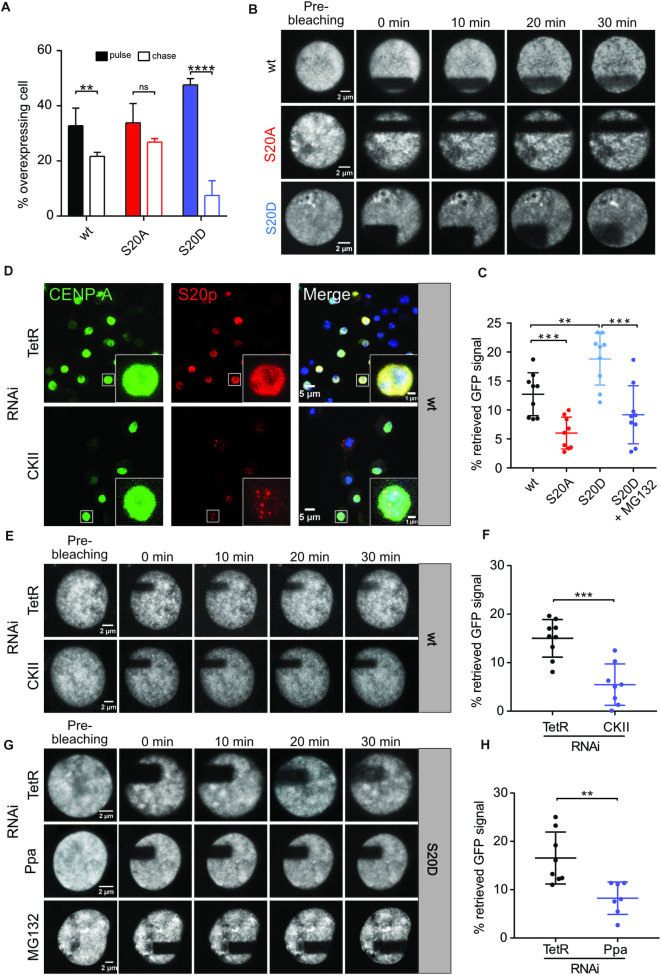
High turn-over of overexpressed phosphomimetic GFP-CENP-A_S20D at non-centromeric chromatin sites. (**A**) Cells were subjected to pulse (8 h)-chase (16 h) GFP-CENP-A expression. The quantification of cells with CENP-A overexpression after pulse and chase periods is shown. A total of 172–679 cells was analyzed. Mean ± SEM values are shown. Statistical significance was calculated by unpaired *t*-test. (B) GFP-CENP-A was overexpressed for 16 h before bleaching as described in Materials and Methods. Images were taken before and after bleaching as indicated. (**C**) Quantification of GFP signal recovery for wt, S20A and S20D mutant CENP-A after bleaching. Intensities of identical squares within (*B*) and outside the bleached area (*NB*) were determined by ImageJ at 0 and 30 min after bleaching to calculate a *B/NB* ratio. The difference between *B/NB (30 min)* and *B/NB (0 min)* corresponds to the % of retrieved GFP signal (*n* = 9 nuclei for each cell line). Mean ± SEM values are shown. Statistical significance was calculated by unpaired *t*-test. (**D**) GFP-CENP-A_wt cells were subjected to RNAi against TetR (control) or CKII before induction of CENP-A overexpression for 16 h and immunofluorescence imaging. Cells were stained for GFP-CENP-A and S20p. Insets are 4x enlargements of indicated nuclei. (**E**) FRAP analysis of cells treated with dsRNA against CKII and TetR. (**F**) Quantification of GFP signals from E. (**G**) FRAP analysis of GFP-CENP-A_S20D overexpressing cells treated with dsRNA against Ppa and TetR. Where indicated, the proteasome inhibitor MG132 was added to GFP-CENP-A_S20D cells 5 h prior to FRAP analysis. (**H**) Quantification of GFP-signals from RNAi experiments shown in G as described for C. **P< 0.01, ***P< 0.001.

Next, we performed pulse-chase expression experiments to monitor protein clearance under overexpression conditions. To this end, GFP-tagged wt and mutant proteins were induced for eight hours, then CuSO_4_ was washed out, and cells were collected for fluorescence microscopy right after CuSO_4_ removal and after additional 16 h cultivation (chase period). Comparison of GFP signals revealed a strong decrease of the fraction of cells showing diffuse nuclear CENP-A_S20D staining, intermediate decrease for CENP-A_wt and almost no decrease for the S20A mutant (Figure 9A, Supplementary Figure S8). To more accurately determine the dynamics of ectopically incorporated CENP-A, we performed FRAP experiments in GFP-CENP-A overexpressing cells. The results demonstrated that GFP-CENP-A_S20D recovered significantly faster at non-centromeric sites than GFP-CENP-A_wt (18% versus 12% recovery). By contrast, recovery of the non-phosphorylatable GFP-CENP-A_S20A was strongly reduced (6%; Figure 9B, C; Supplementary Figure S9A). When we knocked-down CKII in GFP-CENP-A_wt expressing cells, S20 phosphorylation was strongly reduced at non-centromeric sites and only detectable at a few distinct foci, presumably corresponding to the endogenous centromeres (Figure 9D, Supplementary Figure S9B). FRAP analyses with these cells showed a strong reduction of GFP signal recovery similar to that of the S20A mutant (5%; Figure 9E, F) corroborating the important role of S20 phosphorylation. Moreover, we examined, if fast signal recovery of GFP-CENP-A_S20D depends on proteolysis. To this end, we performed FRAP with cells that had been treated with the proteasome inhibitor MG132 for 5 h prior to imaging. Proteasome inhibition clearly and significantly reduced GFP-signal recovery after bleaching (Figure 9C, G). Moreover, signal recovery of S20D CENP-A was reduced upon knock-down of Ppa (Figure 9G, H; Supplementary Figure S9B). These results strongly suggest that signal recovery requires degradation of preexisting protein.

In summary, S20 phosphorylation appears to be necessary for fast removal of aberrantly incorporated CENP-A by proteasomal degradation.

## DISCUSSION

In this study, we examined the functional consequences of CENP-A phosphorylation at the N-terminally located serine 20. Our analyses revealed that though S20 phosphorylation is present in the soluble protein pool, it is particularly enriched in chromatin-bound CENP-A. We identified CKII as the enzyme responsible for S20 phosphorylation. Notably, we found three major functions of S20 phosphorylation: First, it is required for efficient loading/maintenance of CENP-A to the chromatin, second, it regulates the levels of soluble CENP-A by controlling proteolysis upstream of the ubiquitin ligase SCF^Ppa^, and third, it controls the amount of aberrantly incorporated CENP-A in chromatin outside of centromeres.

### Role of S20 phosphorylation in regulating the stability of pre-nucleosomal CENP-A

The tight regulation of CENP-A abundance is a recurrent strategy by which different organisms ensure that CENP-A assembly is restricted to the centromere. Proteolysis of excess amounts of CENP-A by the proteasome has been detected in yeast and flies (66,67) but has not been demonstrated for human cells so far. Consistent with the fact that polyubiquitylation of target proteins is a hallmark of proteasomal degradation, budding yeast CENP-A^Cse4^ was shown to be ubiquitylated by the E3 ligase Psh1 thereby preventing its ectopic incorporation (68,69). Likewise, in *Drosophila*, the ubiquitin ligase adapter F-box protein Ppa is required for ubiquitylation and proteolytic clearance of CENP-A from ectopic sites (59,67). Very recently, the APC/C^Cdh1^ complex has been identified as another E3 ligase regulating CENP-A protein degradation in the fly (70).

Our studies showed that soluble CENP-A becomes substantially more labile when S20 is mutated to the phosphomimetic aspartate, while it displays increased stability compared to wild-type CENP-A when S20 is mutated to the non-phosphorylatable alanine (Figure 5C). Knock-down of Ppa increased the half-lives of wild-type and S20D-mutated but not of S20A-mutated CENP-A. Because substrate recognition of SCF ubiquitin ligases is often determined by phosphorylation of the target protein (61), our results suggest that CENP-A needs to be phosphorylated before it can be targeted for degradation by SCF^Ppa^. Moreno-Moreno *et al.* have shown that Ppa interacts with both the histone fold domain of CENP-A and its N-terminal region (59). Thus, it is conceivable that N-terminal phosphorylation affects the ability of CENP-A to be ubiquitylated by SCF^Ppa^. Notably, when CENP-A is in complex with CAL1, SCF^Ppa^ cannot induce degradation, while APC/C^Cdh1^ can modify CENP-A regardless of its presence in the CAL1 complex (70). Our degradation experiments with CENP-A_S20D/A were performed upon overexpression of CENP-A to allow for detection of soluble protein by western blot. Under these conditions, we observed a clear effect of Ppa knock-down on decay kinetics of the S20D mutant protein (Figure 6) suggesting that most of the detected protein was not in a complex with CAL1 and therefore accessible to SCF^Ppa^. Thus, we conclude that S20 phosphorylation renders CENP-A a substrate for ubiquitylation by SCF^Ppa^, thereby regulating the abundance of free pre-nucleosomal CENP-A. The role for S20 phosphorylation in protein turn-over is further supported by the fact that knock-down of CKII, which removes S20 phosphorylation, also leads to a stabilization of CENP-A (Figure 5). CKII, which we show here to be the kinase responsible for S20 phosphorylation, has also been implicated in CENP-A turn-over in budding yeast. However, in that case, phosphorylation of the E3 ligase Psh1 and not of CENP-A itself promoted CENP-A degradation (58).

### Role of S20 phosphorylation in regulating turn-over of chromatin-bound CENP-A

In contrast to the results obtained for soluble and therefore pre-nucleosomal CENP-A, incorporation into centromeric chromatin seemed to protect the phosphomimetic CENP-A_S20D from proteolysis. This is on one hand reflected in the relative higher abundance of chromatin-bound CENP-A_S20D versus endogenous CENP-A under non-overexpression conditions (Figure 4B, Supplementary Figure S4) and on the other hand, by the fact that under non-overexpression conditions the stability of chromatin (= centromere)-bound CENP-A was high regardless of whether S20 was mutated to A or D or not mutated (Figure 8A, B). By contrast, upon transient overexpression leading to nucleus-wide incorporation of CENP-A, chromatin-bound phosphomimetic S20D was more quickly degraded than the wt and S20A mutant proteins (Figure 8A, B). The increased proteasome-dependent turn-over rate of S20D compared to S20A and wt CENP-A outside of centromeres was further evident from FRAP measurements (Figure 9). Hence, S20 phosphorylation promotes both the maintenance of low levels of pre-nucleosomal CENP-A, which likely contributes to minimizing ectopic incorporation, and the removal of CENP-A from chromatin in situations when it is promiscuously loaded into non-centromeric sites. Somewhat counterintuitively, S20 is also highly phosphorylated at its centromeric location (Figure 1F) although the protein is stable there. Interestingly, knock-down of CKII in CENP-A overexpressing cells reduces the previously uniformly distributed S20 phosphorylation signal to few distinct foci in interphase nuclei (Figure 9D). These foci might correspond to centromeric CENP-A signals, which would further support the idea that S20-phosphorylated CENP-A is stable at centromeres, since the protein must have become phosphorylated before CKII levels decreased. It is possible that binding of CAL1 might mask phosphorylated S20, thereby protecting CENP-A from Ppa-dependent polyubiquitylation and degradation as shown recently by Moreno-Moreno *et al.* in *Drosophila* larvae (70). Centromeric CAL1 might thus allow the accumulation of S20p-CENP-A at centromeres. However, further studies are needed to answer this question. Although currently the purpose of S20-phosphorylation of centromeric CENP-A is unknown, it is likely to have additional roles, e.g. in the establishment of the kinetochore. Indeed, cells expressing the non-phosphorylatable CENP-A S20A protein proliferate more slowly, show an increased mitotic index and higher incidence of mitotic defects in metaphase (Figure 3E, F) suggesting defects in kinetochore formation. Of note, however, the phoshpomimetic S20D protein also causes slight defects in proliferation and mitosis (Figure 3E, F), which suggests the requirement of balanced amounts of phosphorylated S20 for proper function. Alternatively or in addition, S20 phosphorylation might be involved in the maintenance of old CENP-A during the transcription-coupled loading of new CENP-A in mitosis (27–29). Our observation that mitotically arrested cells and cells in G2/M phase exhibit an accumulation of S20-phosphorylated CENP-A compared to asynchronous cell populations (Figure 1E, F) may support such a hypothesis.

### Role of S20 phosphorylation in CENP-A loading

In addition to the observed effects of S20 phosphorylation on CENP-A degradation, our results also suggest that it affects CENP-A loading to the chromatin. This conclusion is supported by the observations that the non-phosphorylatable CENP-A_S20A protein shows significantly lower centromeric steady-state levels in non-overexpression conditions (Figure 3), and that its abundance in the soluble fraction is higher than that of CENP-A_wt and CENP-A_S20D (Figure 4, Supplementary Figure S4). We considered the possibility that S20 phosphorylation plays a role in the interaction with the different preloading complexes. Thus, we performed immunoprecipitation with α-Flag beads with extracts of wt and mutant SF-CENP-A expressing cells and monitored coprecipitation of CAL1, HAT1 and FACT. We did not observe any differences in the binding of the CENP-A mutant proteins to these CENP-A interaction partners (data not shown). Therefore, at this point the exact role of S20 phosphorylation during CENP-A loading remains unclear.

In summary, our study provides multiple lines of evidence for an important role of S20 phosphorylation in controlling restricted incorporation of CENP-A into centromeric chromatin in *Drosophila*. Regulation of the phosphorylation state of S20, e.g. by modulating CKII activity or by the action of an as yet unknown phosphatase, may provide the cells with a means to fine-tune CENP-A levels in order to prevent deleterious loading to extra-centromeric sites.

## Supplementary Material

gkz809_Supplemental_File

## References

[1] Talbert P.B., Henikoff S. Histone variants — ancient wrap artists of the epigenome. Nat. Rev. Mol. Cell Biol. 2010; 11:264–275. 20197778 10.1038/nrm2861

[2] Allshire R.C., Karpen G.H. Epigenetic regulation of centromeric chromatin: old dogs, new tricks. Nat. Rev. Genet. 2008; 9:923–937. 19002142 10.1038/nrg2466 PMC2586333

[3] Schueler M.G., Sullivan B.A. Structural and functional dynamics of human centromeric chromatin. Annu. Rev. Genomics Hum. Genet. 2006; 7:301–313. 16756479 10.1146/annurev.genom.7.080505.115613

[4] Vagnarelli P., Ribeiro S.A., Earnshaw W.C. Centromeres: old tales and new tools. FEBS Lett. 2008; 582:1950–1959. 18435926 10.1016/j.febslet.2008.04.014

[5] Westhorpe F.G., Straight A.F. The centromere: epigenetic control of chromosome segregation during mitosis. Cold Spring Harbor Perspect. Biol. 2014; 7:a015818. 10.1101/cshperspect.a015818 PMC4292168 25414369

[6] Müller S., Almouzni G. A network of players in H3 histone variant deposition and maintenance at centromeres. Biochim. Biophys. Acta. 2014; 1839:241–250. 24316467 10.1016/j.bbagrm.2013.11.008

[7] Chen C.-C., Mellone B.G. Chromatin assembly: journey to the CENter of the chromosome. J. Cell Biol. 2016; 214:13–24. 27377247 10.1083/jcb.201605005 PMC4932374

[8] Barnhart M.C., Kuich P.H.J.L., Stellfox M.E., Ward J.A., Bassett E.A., Black B.E., Foltz D.R. HJURP is a CENP-A chromatin assembly factor sufficient to form a functional de novo kinetochore. J. Cell Biol. 2011; 194:229–243. 21768289 10.1083/jcb.201012017 PMC3144403

[9] Fujita Y., Hayashi T., Kiyomitsu T., Toyoda Y., Kokubu A., Obuse C., Yanagida M. Priming of centromere for CENP-A recruitment by human hMis18alpha, hMis18beta, and M18BP1. Dev. Cell. 2007; 12:17–30. 17199038 10.1016/j.devcel.2006.11.002

[10] Moree B., Meyer C.B., Fuller C.J., Straight A.F. CENP-C recruits M18BP1 to centromeres to promote CENP-A chromatin assembly. J. Cell Biol. 2011; 194:855–871. 21911481 10.1083/jcb.201106079 PMC3207292

[11] Silva M.C.C., Bodor D.L., Stellfox M.E., Martins N.M.C., Hochegger H., Foltz D.R., Jansen L.E.T. Cdk activity couples epigenetic centromere inheritance to cell cycle progression. Dev. Cell. 2012; 22:52–63. 22169070 10.1016/j.devcel.2011.10.014

[12] Wong L.H., Brettingham-Moore K.H., Chan L., Quach J.M., Anderson M.A., Northrop E.L., Hannan R., Saffery R., Shaw M.L., Williams E. et al. Centromere RNA is a key component for the assembly of nucleoproteins at the nucleolus and centromere. Genome Res. 2007; 17:1146–1160. 17623812 10.1101/gr.6022807 PMC1933521

[13] Ideue T., Cho Y., Nishimura K., Tani T. Involvement of satellite I noncoding RNA in regulation of chromosome segregation. Genes Cells. 2014; 19:528–538. 24750444 10.1111/gtc.12149

[14] Chan F.L., Marshall O.J., Saffery R., Kim B.W., Earle E., Choo K.H.A., Wong L.H. Active transcription and essential role of RNA polymerase II at the centromere during mitosis. Proc. Natl. Acad. Sci. U.S.A. 2012; 109:1979–1984. 22308327 10.1073/pnas.1108705109 PMC3277563

[15] Blower M.D. Centromeric transcription regulates Aurora-B localization and activation. Cell Rep. 2016; 15:1624–1633. 27184843 10.1016/j.celrep.2016.04.054 PMC4880503

[16] McNulty S.M., Sullivan L.L., Sullivan B.A. Human Centromeres Produce Chromosome-Specific and Array-Specific Alpha Satellite Transcripts that Are Complexed with CENP-A and CENP-C. Dev. Cell. 2017; 42:226–240. 28787590 10.1016/j.devcel.2017.07.001 PMC5568664

[17] Schuh M., Lehner C.F., Heidmann S. Incorporation of Drosophila CID/CENP-A and CENP-C into centromeres during early embryonic anaphase. Curr. Biol. 2007; 17:237–243. 17222555 10.1016/j.cub.2006.11.051

[18] Mellone B.G., Grive K.J., Shteyn V., Bowers S.R., Oderberg I., Karpen G.H. Assembly of Drosophila centromeric chromatin proteins during mitosis. PLoS Genet. 2011; 7:e1002068. 21589899 10.1371/journal.pgen.1002068 PMC3093364

[19] Goshima G., Wollman R., Goodwin S.S., Zhang N., Scholey J.M., Vale R.D., Stuurman N. Genes required for mitotic spindle assembly in Drosophila S2 cells. Science. 2007; 316:417–421. 17412918 10.1126/science.1141314 PMC2837481

[20] Erhardt S., Mellone B.G., Betts C.M., Zhang W., Karpen G.H., Straight A.F. Genome-wide analysis reveals a cell cycle-dependent mechanism controlling centromere propagation. J. Cell Biol. 2008; 183:805–818. 19047461 10.1083/jcb.200806038 PMC2592830

[21] Chen C.-C., Dechassa M.L., Bettini E., Ledoux M.B., Belisario C., Heun P., Luger K., Mellone B.G. CAL1 is the Drosophila CENP-A assembly factor. J. Cell Biol. 2014; 204:313–329. 24469636 10.1083/jcb.201305036 PMC3912524

[22] Schittenhelm R.B., Althoff F., Heidmann S., Lehner C.F. Detrimental incorporation of excess Cenp-A/Cid and Cenp-C into Drosophila centromeres is prevented by limiting amounts of the bridging factor Cal1. J. Cell Sci. 2010; 123:3768–3779. 20940262 10.1242/jcs.067934

[23] Boltengagen M., Huang A., Boltengagen A., Trixl L., Lindner H., Kremser L., Offterdinger M., Lusser A. A novel role for the histone acetyltransferase Hat1 in the CENP-A/CID assembly pathway in Drosophila melanogaster. Nucleic Acids Res. 2016; 44:2145–2159. 26586808 10.1093/nar/gkv1235 PMC4797270

[24] Furuyama T., Henikoff S. Biotin-tag affinity purification of a centromeric nucleosome assembly complex. Cell Cycle. 2006; 5:1269–1274. 16775420 10.4161/cc.5.12.2889

[25] Barth T.K., Schade G.O.M., Schmidt A., Vetter I., Wirth M., Heun P., Imhof A., Thomae A.W. Identification of drosophila centromere associated proteins by quantitative affinity purification-mass spectrometry. Data Brief. 2015; 4:544–550. 26306323 10.1016/j.dib.2015.07.016 PMC4536286

[26] Campos E.I., Fillingham J., Li G., Zheng H., Voigt P., Kuo W.-H.W., Seepany H., Gao Z., Day L.A., Greenblatt J.F. et al. The program for processing newly synthesized histones H3.1 and H4. Nat. Struct. Mol. Biol. 2010; 17:1343. 20953179 10.1038/nsmb.1911 PMC2988979

[27] Bobkov G.O.M., Gilbert N., Heun P. Centromere transcription allows CENP-A to transit from chromatin association to stable incorporation. J. Cell Biol. 2018; 217:1957–1972. 29626011 10.1083/jcb.201611087 PMC5987708

[28] Chen C.-C., Bowers S., Lipinszki Z., Palladino J., Trusiak S., Bettini E., Rosin L., Przewloka M.R., Glover D.M., O’Neill R.J. et al. Establishment of centromeric chromatin by the CENP-A assembly factor CAL1 requires FACT-mediated transcription. Dev. Cell. 2015; 34:73–84. 26151904 10.1016/j.devcel.2015.05.012 PMC4495351

[29] Rošić S., Köhler F., Erhardt S. Repetitive centromeric satellite RNA is essential for kinetochore formation and cell division. J. Cell Biol. 2014; 207:335–349. 25365994 10.1083/jcb.201404097 PMC4226727

[30] Rošić S., Erhardt S. No longer a nuisance: long non-coding RNAs join CENP-A in epigenetic centromere regulation. Cell Mol. Life Sci. 2016; 73:1387–1398. 26748759 10.1007/s00018-015-2124-7 PMC11108473

[31] Talbert P.B., Henikoff S. Transcribing centromeres: Noncoding RNAs and Kinetochore Assembly. Trends Genet. 2018; 34:587–599. 29871772 10.1016/j.tig.2018.05.001

[32] Sharma A.B., Dimitrov S., Hamiche A., Van Dyck E. Centromeric and ectopic assembly of CENP-A chromatin in health and cancer: old marks and new tracks. Nucleic Acids Res. 2018; 91:313. 10.1093/nar/gky1298 PMC6379705 30590707

[33] Malik H.S., Henikoff S. Adaptive evolution of Cid, a centromere-specific histone in Drosophila. Genetics. 2001; 157:1293–1298. 11238413 10.1093/genetics/157.3.1293 PMC1461554

[34] Xu Y.M., Du J.Y., Lau A.T.Y. Posttranslational modifications of human histone H3: An update. Proteomics. 2014; 14:2047–2060. 25044606 10.1002/pmic.201300435

[35] Srivastava S., Foltz D.R. Posttranslational modifications of CENP-A: marks of distinction. Chromosoma. 2018; 127:1–12. 29569072 10.1007/s00412-018-0665-x PMC6082721

[36] Bailey A.O., Panchenko T., Sathyan K.M., Petkowski J.J., Pai P.-J., Bai D.L., Russell D.H., Macara I.G., Shabanowitz J., Hunt D.F. et al. Posttranslational modification of CENP-A influences the conformation of centromeric chromatin. Proc. Natl. Acad. Sci. U.S.A. 2013; 110:11827–11832. 23818633 10.1073/pnas.1300325110 PMC3718089

[37] Sathyan K.M., Fachinetti D., Foltz D.R. α-amino trimethylation of CENP-A by NRMT is required for full recruitment of the centromere. Nat. Commun. 2017; 8:14678. 28266506 10.1038/ncomms14678 PMC5343448

[38] Bui M., Dimitriadis E.K., Hoischen C., An E., Quénet D., Giebe S., Nita-Lazar A., Diekmann S., Dalal Y. Cell-cycle-dependent structural transitions in the human CENP-A nucleosome in vivo. Cell. 2012; 150:317–326. 22817894 10.1016/j.cell.2012.05.035 PMC3592566

[39] Bui M., Pitman M., Nuccio A., Roque S., Donlin-Asp P.G., Nita-Lazar A., Papoian G.A., Dalal Y. Internal modifications in the CENP-A nucleosome modulate centromeric dynamics. Epigenet. Chromatin. 2017; 10:17. 10.1186/s13072-017-0124-6 PMC5379712 28396698

[40] Niikura Y., Kitagawa R., Kitagawa K. CENP-A Ubiquitylation Is Required for CENP-A Deposition at the centromere. Dev Cell. 2017; 40:7–8. 28073011 10.1016/j.devcel.2016.12.020 PMC6667218

[41] Fukagawa T. Critical histone post-translational modifications for centromere function and propagation. Cell Cycle. 2017; 16:1259–1265. 28598241 10.1080/15384101.2017.1325044 PMC5531634

[42] Zeitlin S.G., Shelby R.D., Sullivan K.F. CENP-A is phosphorylated by Aurora B kinase and plays an unexpected role in completion of cytokinesis. J. Cell Biol. 2001; 155:1147–1157. 11756469 10.1083/jcb.200108125 PMC2199334

[43] Kunitoku N., Sasayama T., Marumoto T., Zhang D., Honda S., Kobayashi O., Hatakeyama K., Ushio Y., Saya H., Hirota T. CENP-A phosphorylation by Aurora-A in prophase is required for enrichment of Aurora-B at inner centromeres and for kinetochore function. Dev. Cell. 2003; 5:853–864. 14667408 10.1016/s1534-5807(03)00364-2

[44] Goutte-Gattat D., Shuaib M., Ouararhni K., Gautier T., Skoufias D.A., Hamiche A., Dimitrov S. Phosphorylation of the CENP-A amino-terminus in mitotic centromeric chromatin is required for kinetochore function. Proc. Natl. Acad. Sci. U.S.A. 2013; 110:8579–8584. 23657009 10.1073/pnas.1302955110 PMC3666736

[45] Takada M., Zhang W., Suzuki A., Kuroda T.S., Yu Z., Inuzuka H., Gao D., Wan L., Zhuang M., Hu L. et al. FBW7 Loss promotes chromosomal instability and tumorigenesis via cyclin E1/CDK2-mediated phosphorylation of CENP-A. Cancer Res. 2017; 77:4881–4893. 28760857 10.1158/0008-5472.CAN-17-1240 PMC5743019

[46] Hu H., Liu Y., Wang M., Fang J., Huang H., Yang N., Li Y., Wang J., Yao X., Shi Y. et al. Structure of a CENP-A-histone H4 heterodimer in complex with chaperone HJURP. Genes Dev. 2011; 25:901–906. 21478274 10.1101/gad.2045111 PMC3084024

[47] Yu Z., Zhou X., Wang W., Deng W., Fang J., Hu H., Wang Z., Li S., Cui L., Shen J. et al. Dynamic phosphorylation of CENP-A at Ser68 orchestrates its cell-cycle-dependent deposition at centromeres. Dev. Cell. 2015; 32:68–81. 25556658 10.1016/j.devcel.2014.11.030

[48] Wang K., Yu Z., Liu Y., Li G. Ser68 phosphorylation ensures accurate cell-cycle-dependent CENP-A deposition at centromeres. Dev. Cell. 2017; 40:5–6. 28073010 10.1016/j.devcel.2016.12.015

[49] Barra V., Logsdon G.A., Scelfo A., Hoffmann S., Hervé S., Aslanian A., Nechemia-Arbely Y., Cleveland D.W., Black B.E., Fachinetti D. Phosphorylation of CENP-A on serine 7 does not control centromere function. Nat. Commun. 2019; 10:175. 30635586 10.1038/s41467-018-08073-1 PMC6329807

[50] Fachinetti D., Logsdon G.A., Abdullah A., Selzer E.B., Cleveland D.W., Black B.E. CENP-A Modifications on Ser68 and Lys124 Are Dispensable for establishment, maintenance, and Long-Term function of human centromeres. Dev. Cell. 2017; 40:104–113. 28073008 10.1016/j.devcel.2016.12.014 PMC5235356

[51] Iwaki T., Castellino F.J. A single plasmid transfection that offers a significant advantage associated with puromycin selection in Drosophila Schneider S2 cells expressing heterologous proteins. Cytotechnology. 2008; 57:45–49. 19003171 10.1007/s10616-008-9129-0 PMC2553637

[52] Fleming S.L., Rieder C.L. Flattening Drosophila cells for high-resolution light microscopic studies of mitosis in vitro. Cell Motil. Cytoskeleton. 2003; 56:141–146. 14569594 10.1002/cm.10143

[53] Blom N., Sicheritz-Pontén T., Gupta R., Gammeltoft S., Brunak S. Prediction of post-translational glycosylation and phosphorylation of proteins from the amino acid sequence. Proteomics. 2004; 4:1633–1649. 15174133 10.1002/pmic.200300771

[54] Zasadzińska E., Barnhart-Dailey M.C., Kuich P.H.J.L., Foltz D.R. Dimerization of the CENP-A assembly factor HJURP is required for centromeric nucleosome deposition. EMBO J. 2013; 32:2113–2124. 23771058 10.1038/emboj.2013.142 PMC3730228

[55] Au W.-C., Crisp M.J., DeLuca S.Z., Rando O.J., Basrai M.A. Altered dosage and mislocalization of histone H3 and Cse4p lead to chromosome loss in Saccharomyces cerevisiae. Genetics. 2008; 179:263–275. 18458100 10.1534/genetics.108.088518 PMC2390605

[56] Heun P., Erhardt S., Blower M.D., Weiss S., Skora A.D., Karpen G.H. Mislocalization of the Drosophila centromere-specific histone CID promotes formation of functional ectopic kinetochores. Dev. Cell. 2006; 10:303–315. 16516834 10.1016/j.devcel.2006.01.014 PMC3192491

[57] Gowda C., Song C., Kapadia M., Payne J.L., Hu T., Ding Y., Dovat S. Regulation of cellular proliferation in acute lymphoblastic leukemia by Casein Kinase II (CK2) and Ikaros. Adv. Biol. Regul. 2017; 63:71–80. 27666503 10.1016/j.jbior.2016.09.003 PMC6053069

[58] Hewawasam G.S., Mattingly M., Venkatesh S., Zhang Y., Florens L., Workman J.L., Gerton J.L. Phosphorylation by casein kinase 2 facilitates Psh1 protein-assisted degradation of Cse4 protein. J. Biol. Chem. 2014; 289:29297–29309. 25183013 10.1074/jbc.M114.580589 PMC4200280

[59] Moreno-Moreno O., Medina-Giró S., Torras-Llort M., Azorín F. The F box protein partner of paired regulates stability of Drosophila centromeric histone H3, CenH3(CID). Curr. Biol. 2011; 21:1488–1493. 21871803 10.1016/j.cub.2011.07.041

[60] Ho M.S., Ou C., Chan Y.R., Chien C.T., Pi H. The utility F-box for protein destruction. Cell Mol. Life Sci. 2008; 65:1977–2000. 18344020 10.1007/s00018-008-7592-6 PMC11131715

[61] Vodermaier H.C. APC/C and SCF: controlling each other and the cell cycle. Curr. Biol. 2004; 14:R787–R796. 15380093 10.1016/j.cub.2004.09.020

[62] Gonzalez M., He H., Dong Q., Sun S., Li F. Ectopic centromere nucleation by CENP–a in fission yeast. Genetics. 2014; 198:1433–1446. 25298518 10.1534/genetics.114.171173 PMC4256763

[63] Shrestha R.L., Ahn G.S., Staples M.I., Sathyan K.M., Karpova T.S., Foltz D.R., Basrai M.A. Mislocalization of centromeric histone H3 variant CENP-A contributes to chromosomal instability (CIN) in human cells. Oncotarget. 2017; 8:46781–46800. 28596481 10.18632/oncotarget.18108 PMC5564523

[64] Lacoste N., Woolfe A., Tachiwana H., Garea A.V., Barth T., Cantaloube S., Kurumizaka H., Imhof A., Almouzni G. Mislocalization of the centromeric histone variant CenH3/CENP-A in human cells depends on the chaperone DAXX. Mol. Cell. 2014; 53:631–644. 24530302 10.1016/j.molcel.2014.01.018

[65] Chang C.-H., Chavan A., Palladino J., Wei X., Martins N.M.C., Santinello B., Chen C.-C., Erceg J., Beliveau B.J., Wu C.-T. et al. Islands of retroelements are major components of Drosophila centromeres. PLoS Biol. 2019; 17:e3000241. 31086362 10.1371/journal.pbio.3000241 PMC6516634

[66] Collins K.A., Furuyama S., Biggins S. Proteolysis contributes to the exclusive centromere localization of the yeast Cse4/CENP-A histone H3 variant. Curr. Biol. 2004; 14:1968–1972. 15530401 10.1016/j.cub.2004.10.024

[67] Moreno-Moreno O., Torras-Llort M., Azorín F. Proteolysis restricts localization of CID, the centromere-specific histone H3 variant of Drosophila, to centromeres. Nucleic Acids Res. 2006; 34:6247–6255. 17090596 10.1093/nar/gkl902 PMC1693906

[68] Hewawasam G., Shivaraju M., Mattingly M., Venkatesh S., Martin-Brown S., Florens L., Workman J.L., Gerton J.L. Psh1 is an E3 ubiquitin ligase that targets the centromeric histone variant Cse4. Mol. Cell. 2010; 40:444–454. 21070970 10.1016/j.molcel.2010.10.014 PMC2998187

[69] Ranjitkar P., Press M.O., Yi X., Baker R., MacCoss M.J., Biggins S. An E3 ubiquitin ligase prevents ectopic localization of the centromeric histone H3 variant via the centromere targeting domain. Mol. Cell. 2010; 40:455–464. 21070971 10.1016/j.molcel.2010.09.025 PMC2995698

[70] Moreno-Moreno O., Torras-Llort M., Azorín F. The E3-ligases SCFPpa and APC/CCdh1 co-operate to regulate CENP-ACID expression across the cell cycle. Nucleic Acids Res. 2019; 9:923. 10.1093/nar/gkz060 PMC6468245 30753559

