# Supplementary material for: Phosphorylation of Drosophila CENP-A on serine 20 regulates protein turn-over and centromere-specific loading
Source: Nucleic Acids Res. 2019 Sep 19;47(20):10754–70. doi: 10.1093/nar/gkz809 (PMC6847487; doi:10.1093/nar/gkz809)
Supplement: gkz809_Supplemental_File [file gkz809_supplemental_file.pdf]

Supplementary Material

Phosphorylation of *Drosophila* CENP-A on serine 20 regulates protein turn-over and centromere-specific loading

Anming Huang, Leopold Kremser, Fabian Schuler, Doris Wilflingseder, Herbert Lindner, Stephan Geley, Alexandra Lusser

Supplementary Materials and Methods…………………………………………..p2-4
Supplementary References………………………………………………………...p5
Supplementary Figures 1-11…………………………………………………….p6-17

SUPPLEMENTARY MATERIALS AND METHODS

Antibody validation

Specificity tests for the anti-S20p antibody were performed by ELISA and by western blotting of S2 whole cell extracts or of bacterially expressed and purified full-length *Drosophila* CENP-A as a negative control (Fig. S1B). For the latter, bacterially expressed His-tagged CENP-A (1) was purified under denaturing conditions on Ni^{2+}-NTA beads (Qiagen) following manufacturer's instructions. The purified protein was subjected to western blotting and detection with α-CENP-A (1:2,000; Abcam ab10887) or α-S20p (1:1,000; 1:100,000) antibody (Fig. S1B).

Protein extract preparation

To examine the levels of endogenous CENP-A or of induced or non-induced transgenic CENP-A in different cellular compartments, cytoplasmic, nuclear and chromatin extracts were prepared exactly as described before (1). For the analysis of posttranslational modifications and of degradation kinetics of chromatin-bound SF-CENP-A, chromatin extract preparation was modified as follows: 10^9 cells were harvested, washed with PBS and lysed in RIPA buffer (10 mM Tris-HCl, pH 8.0, 1 mM EDTA, 1% Triton X-100, 0.1% sodium deoxycholate, 0.1% SDS, 140 mM NaCl, 1 mM PMSF, 1× Protease inhibitor cocktail) by grinding with a small pestle for 5 minutes on ice. Chromatin and chromatin binding proteins were collected by centrifugation at 20,000×g for 15 minutes at 4 °C. To the pellet, 30 µl 1×Laemmli sample buffer was added followed by sonication at 4℃ in a Bioruptor instrument (Diagenode) for 30 minutes (30 seconds on, 30 seconds off; setting "high"). The extract was cleared by centrifugation as above. To examine degradation of total CENP-A, whole cell protein extracts (WCE) were prepared: Typically, 10^7 cells were harvested, washed in PBS and lysed and extracted by the addition of 1 volume 2×SDS loading buffer to the pellet followed by sonication as described above. Subsequently, cellular debris was removed by centrifugation at 20,000×g for 15 minutes at RT.

Total protein extracts from *Drosophila* embryos collected at 0-12 h after egg deposition (~50 µl packed volume) were prepared as follows: embryos were dechorionated with 50% bleach, followed by rinsing in water. Five volumes of 1× SDS loading buffer were added, embryos were ground with a small pestle for 5 min and subsequently subjected to sonication and centrifugation as described above. Without disturbing the lipid layer on top, the supernatant was carefully transferred to a fresh tube and centrifuged again. This was repeated three times.

Protein extracts from FACS-sorted cells were prepared as follows: 3.2×10^7 cells each in G1, S and G2/M phase were sorted into tubes containing 5x RIPA buffer to immediately lyse the cells and prevent further cell cycle progression. Samples were centrifuged for 30 min at 20,000×g, the supernatant was removed and the pellet containing chromatin and chromatin-bound proteins was suspended in 50 µl 1× SDS loading buffer. Subsequent sonication and centrifugation were carried out as described above.

Western blotting

Protein samples were separated on 10-12% SDS-PAGE gels and blotted to nitrocellulose membrane. To detect non-overexpressed CENP-A in extracts of soluble protein, extracts were concentrated by vacuum evaporation before loading to the gel. Membranes were blocked in 5% milk in PBS and incubated overnight at 4°C with primary antibodies diluted in 5% milk/PBS followed by washing in PBS

and incubation in HRP-coupled secondary antibody. Signal development was carried out by incubation with ECL (GE Healthcare) followed by detection using a Fusion-SL 3500-WL system (PeqLab) and Fusion software (version 15.17). The following antibodies were used: mouse α-Flag (1:5,000; Proteintech 20543-1-AP), rabbit α-CENP-A (*Drosophila*) (1:2,000; Abcam ab10887), rabbit α-H3 (1:20,000; Millipore 07-690), mouse α-tubulin (1:10,000; Sigma T5168), rabbit α-CENP-A-S20p (1:1,000; this study). Secondary anti-rabbit and anti-mouse IgG antibodies (Sigma) coupled to horseradish peroxidase (HRP) were used at 1:10,000.

Immunofluorescence microscopy

S2 cells were settled down on cover slips for 30 min and fixed with 3.7% paraformaldehyde in PBS/0.3% Triton-X100 for 12 min. After blocking with 5% bovine serum albumin (BSA) in PBS for 60 min, the cells were incubated at 4°C overnight with primary antibodies diluted in 5% BSA/PBS, washed 3 times (10 minutes each) with PBS, followed by incubation with secondary anti-mouse or anti-rabbit antibodies coupled to Alexa Fluor 488 or 594 (Invitrogen) for 60 min at RT. DNA was stained with 5 µg/ml DAPI (Roche) in PBS followed by three washes in PBS. Cells were mounted in Vectashield and imaged on a Leica TCS SP5 microscope. To analyze mitotic chromosomes of S2 cells, cells were resuspended in 10 ml cold 75 mM KCl and kept on ice for 10 min. Cells were centrifuged at 1,000 rpm at 4°C for 5 min and carefully resuspended in 1 ml of 75 mM KCl. 0.1 ml of this cell suspension was added to a well of a live cell imaging chambers (µ-Slide 8 well, ibidi) and centrifuged at 4,000 rpm at 4°C for 5 min. Supernatant was aspirated, cells were fixed and further processed as described above. The following primary antibodies were used: α-CENP-A (1:500; Abcam ab10887), α-CENP-C (1:500; kind gift from Dr. Christian Lehner), α-CENP-A-S20p (1:200, home-made), α-tubulin (1:2,000, Sigma T5168).

FRAP experiments

For FRAP experiments, GFP-CENP-A expressing S2 cells were either directly used (no overexpression; Fig. 8C) or induced by 0.5 mM $CuSO_4$ for 16 h. Cells were allowed to attach to coverslips for 10 min, and subsequently overlaid with agarose sheets according to the method by (2). Briefly, 2% low melting point agarose was melted in S2 cell culture medium, the liquid agarose was pipetted onto coverslips and immediately covered by another coverslip to form a sandwich. After solidification of the agarose, one coverslip was carefully removed with a tweezer, and the agarose-containing coverslip was placed face-down onto the cells. The sandwich was inverted, placed onto a glass slide so that the cells face upward and sealed onto the glass slide with bee wax. FRAP experiments were performed on a Leica TCS SP5 instrument using a 63x/1.40 oil objective. Nuclei showing strong ectopic incorporation of GFP-CENP-A were selected for photobleaching of the GFP signal employing the built-in FRAP-wizard function of the LAS AF software using an argon laser (80% laser power; 10x 1.293 s/frame). Image stacks of 10-20 z-sections of 0.13 µm were captured before, right after bleaching and at 10, 20 and 30 min after bleaching. Four sections each were merged for analysis. To determine signal recovery, fluorescence intensity was measured for a defined area (fixed size rectangle and fixed location) within (*B*) and outside (*NB*) the bleached region of each nucleus at 0 and 30 min after bleaching by ImageJ software (version 1.5). The *B/NB* ratio was calculated and the difference between *B/NB (30 min)* and *B/NB (0 min)* corresponds to the % of retrieved GFP signal.

RNAi experiments

dsRNA probes against 15 different *Drosophila* kinases, Ppa and CENP-A were selected using the algorithm at DRSC/TRiP Functional Genomics Resources (https://fgr.hms.harvard.edu/fly-cell-based-rnai). In addition, nucleotide region 48-573 of the coding sequence of the bacterial Tet repressor (TetR) was chosen as a negative control. Generation of double-stranded RNA probes and RNAi treatment were performed exactly as described previously (1). Briefly, 10^7 S2 cells were washed twice with 10 ml serum-free medium followed by incubation for 30 minutes in 1 ml serum-free medium containing 10 µg of dsRNA before 2 ml of serum-containing medium was added. Cells were treated 5 times at every other day to silence endogenous CENP-A and twice at every other day for Ppa and kinases. Primer sequences used for the generation of dsRNA templates are available upon request. Knock-down efficiency was evaluated by reverse-transcription quantitative PCR (RT-qPCR) on the last day of treatment.

Reverse transcription real time PCR

Total RNA was extracted from $5x10^6$ S2 cells using innuPREP RNA Mini Kit (Analytik Jena), reverse transcribed by GoScript™ Reverse Transcriptase (Promega) and qPCR was performed in a StepOnePlus™ Real-Time PCR System (Applied Biosystems) with Luna® Universal qPCR mix (NEB). Transcript levels were normalized to Gapdh. Gene-specific primers are available upon request.

FACS Analysis

To determine cell cycle profiles, 10^5-10^6 S2 cells were harvested by centrifugation, resuspended in 300 µl PBS and fixed by adding 700 µl cold ethanol absolute followed by careful vortexing. Cells were incubated on ice for 30 min, centrifuged at 300×g for 5 min and resuspended in 1 ml PBS containing 0.1 mg/ml RNase A and 50 µg/ml propidium iodide (PI). After incubation at 37°C for 30 min, FACS analysis was performed on an LSR-Fortessa (BD) instrument and analyzed using FlowJo® v10 software. To determine the percentage of mitotic cells in wt and mutant cell lines, 5×10^5 cells each were fixed in 1 ml ice-cold 70% ethanol at -20°C for 30 min. Cells were washed 3 times with PBS, then resuspended in 300 µl PBS/0.25% Triton X-100 and incubated on ice for 20 min. Following one wash in 1% BSA/PBS, cells were resuspended in 1% BSA/PBS containing Alexa Fluor® 488-coupled α-Histone H3 Phospho (Ser10) antibody (1:50; Biolegend) and incubated for 60 min at RT. After three washes in 1% BSA/PBS, DNA was stained by addition of 5 µg/ml DAPI and samples were subjected to FACS analysis as above. For live cell sorting, 3.2×10^6 cells were stained with 5 µM Hoechst dye prior to sorting according to their DNA content.

SUPPLEMENTARY REFERENCES

1. Boltengagen,M., Huang,A., Boltengagen,A., Trixl,L., Lindner,H., Kremser,L., Offterdinger,M. and Lusser,A. (2016) A novel role for the histone acetyltransferase Hat1 in the CENP-A/CID assembly pathway in Drosophila melanogaster. *Nucleic Acids Res*, **44**, 2145–2159.

2. Fleming,S.L. and Rieder,C.L. (2003) Flattening Drosophila cells for high-resolution light microscopic studies of mitosis in vitro. *Cell Motil Cytoskeleton*, **56**, 141–146.

SUPPLEMENTARY FIGURES

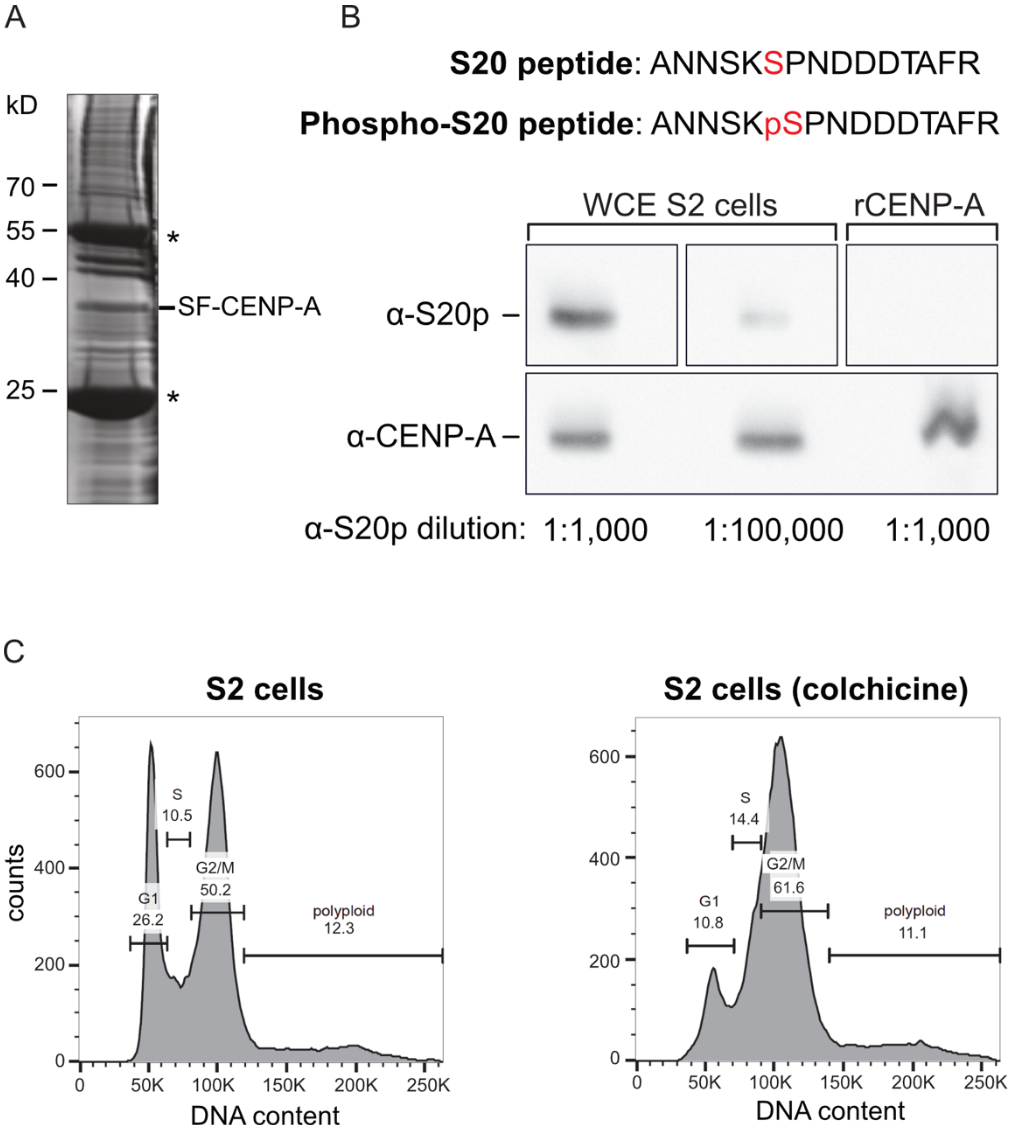

Supplementary Fig. S1. **A** Coomassie-stained gel of SF-CENP-A enriched by anti-Flag purification from chromatin extracts for mass spectrometry analysis. Asterisks denote heavy and light chain IgG bands. **B** Validation of α-S20p antibody. Affinity purified α-S20p antibodies were tested on western blots at the indicated dilutions. The sequences of the peptides used to raise the antibodies (Phospho-S20 peptide) and to negatively purify the sera (S20 peptide) are shown on top. Protein samples were whole cell extract (WCE) from S2 cells and purified His-CENP-A expressed in bacteria. Samples were loaded in duplicates on the same gel, and after blotting, the membrane was cut in half for parallel detection using α-S20p or α-CENP-A (1:1,000) antibodies. Both α-S20p dilutions gave signals at the correct size (27 kDa). By contrast, no signal was detected when the S20p antibody was incubated with bacterially produced and thus unmodified CENP-A confirming the specificity of the antibody for the phosphorylated CENP-A S20 epitope. Detection of the same samples with the α-CENP-A antibody showed that similar amounts of CENP-A were present in all samples. **C** Distribution of cell cycle stages in S2 cells and in S2 cells treated with colchicine to cause mitotic arrest. FACS profiles of propidium iodide stained cells are shown. “G1, S, G2/M” denote the cell cycle stages in the graphs with numbers representing % of cells in the respective stage. Colchicine treatment results in strong arrest in G2/M phase of the cell cycle.

A

B

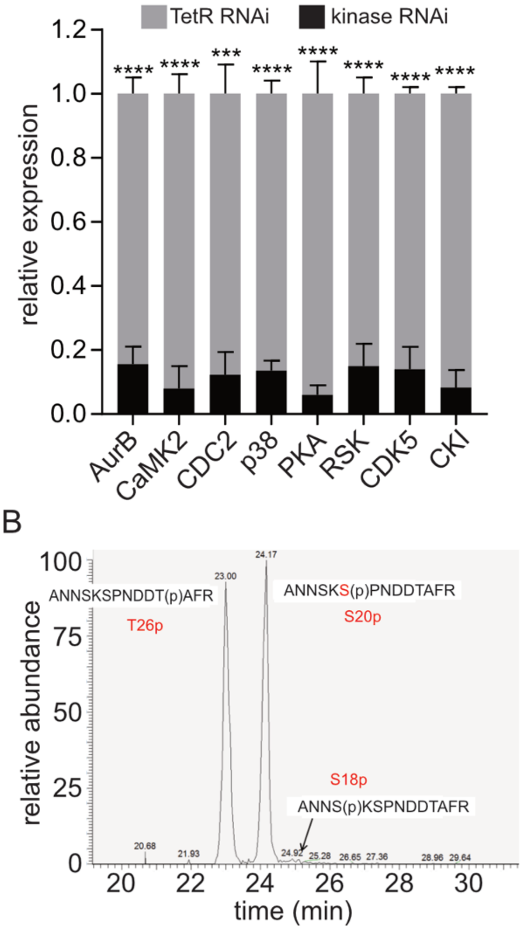

Supplementary Fig. S2. A Reverse-transcription real-time PCR analysis of knock-down efficiency of the indicated kinases in cells treated with the respective dsRNAs or with control TetR dsRNA. Values were normalized against Gapdh. Results are shown relative to the values of the respective TetR control sample, which was set to 1. Mean ± SEM of three biological replicates is shown. Statistical significance was determines by unpaired t-test. ****p<0.0001. **B** Nano-LC MS analysis of a S20-spanning peptide subjected to *in vitro* phosphorylation by CKII. Extracted ion chromatogram of peptide ANNSKSPNDDDTAFR mono-phosphorylated on three different sites (S18, S20, T26).

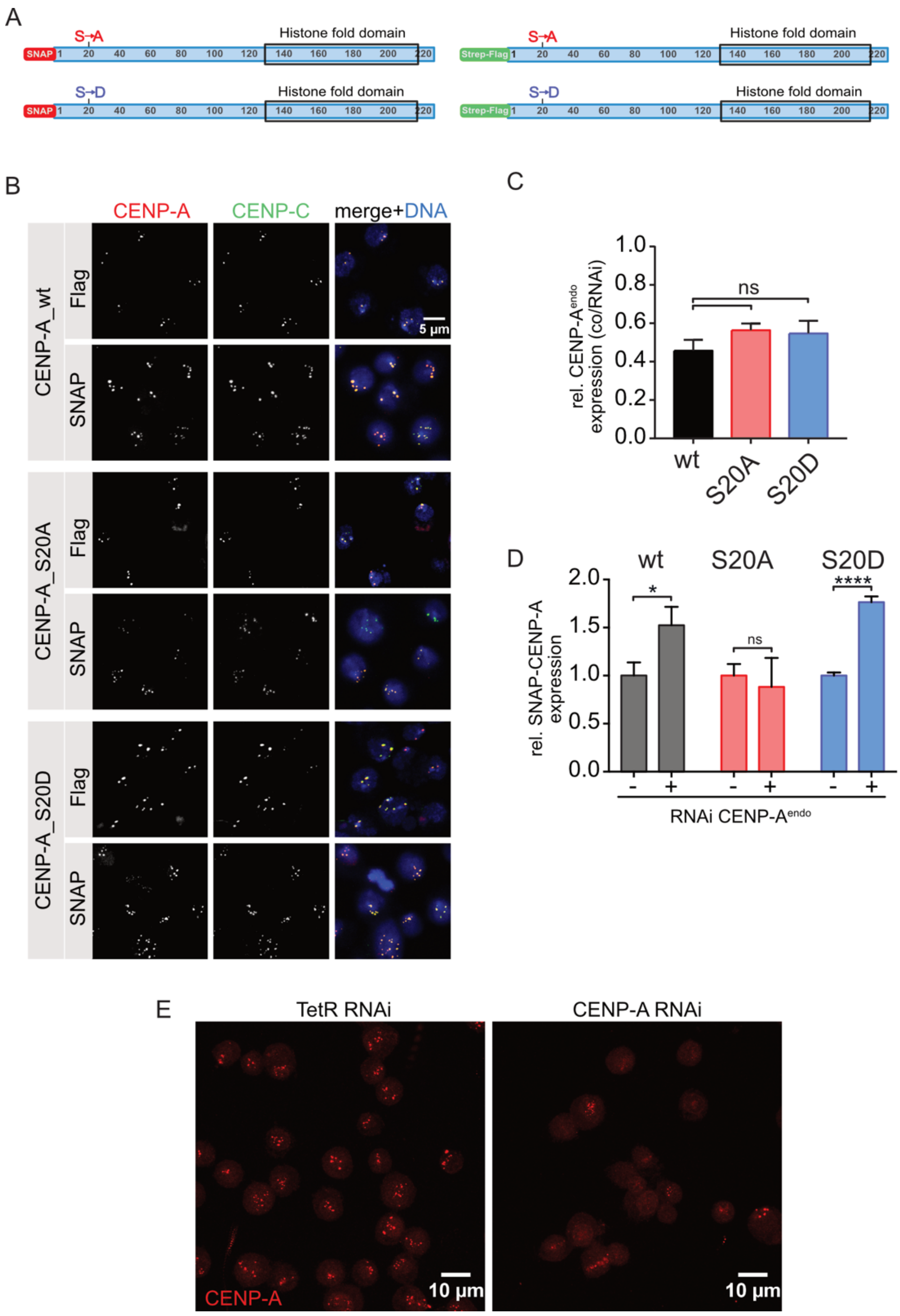

Supplementary Fig. S3. **A** Schematic depiction of the ectopically expressed CENP-A proteins mutated at S20 either fused to an N-terminal SNAP-tag (*left*) or a Strep-Flag-tag (*right*). **B** Localization of Strep-Flag and SNAP-tagged proteins to centromeres in the absence of $CuSO_4$ induction. CENP-A was detected by α-Flag or TMR (SNAP) staining, respectively. α-CENP-C antibodies were used to visualize centromeres. **C** RNAi against CENP-A^{endo} resulted in 40-60% decrease of endogenous CENP-A mRNA expression. ΔCt values of CENP-A RNAi samples were normalized against control samples treated with dsRNA against TetR. Internal reference gene was Gapdh. The different cell lines are indicated. **D** RNAi does not negatively affect transgenic CENP-A transcription. RT-qPCR to determine the effect of CENP-A^{endo} RNAi on transgenic CENP-A levels. ΔCt values of S-CENP-A_wt, S-CENP-A_S20A and S-CENP-A_S20D in samples treated with CENP-A^{endo} RNAi were normalized to ΔCt values of control samples. Means ± SEM of three independent experiments are shown in **C** and **D.** Statistically significance was determined by unpaired t-test. *p<0.05, ****p<0.0001, ns, not significant. **E** Anti-CENP-A staining in untransfected S2 cells subjected either to control RNAi (TetR) or CENP-A RNAi for 10 days.

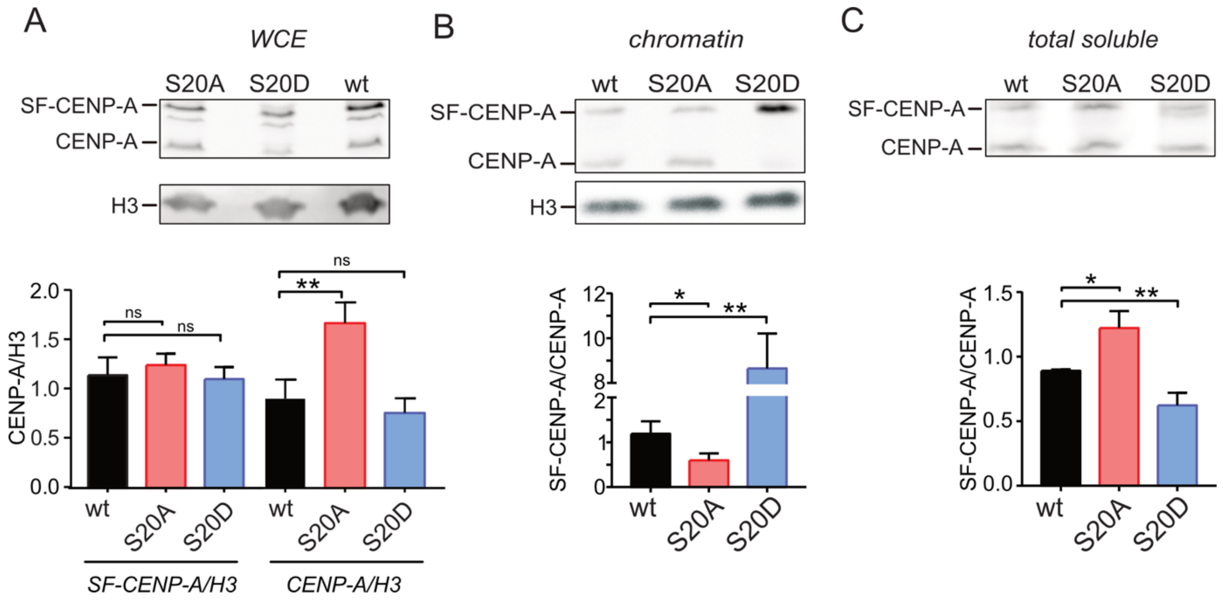

Supplementary Fig. S4. Phosphomimetic CENP-A_S20D is enriched in chromatin but depleted in the soluble fraction. **A** *Upper panel:* Representative western blot of whole cell extract (WCE) from cells expressing Strep-Flag-tagged (SF) wt, S20A or S20D CENP-A, respectively, without induction by $CuSO_4$. α-CENP-A antibody detects transgenic and endogenous CENP-A. α-H3 antibody was used to control for equal loading. *Lower panel:* Quantification of SF-CENP-A and endogenous CENP-A western blot signals. Values were normalized to H3 and are shown as mean ± SEM of three independent experiments. Statistical significance was determined by unpaired t-test with a significance threshold of $p<0.05$. **B** *Upper panel*: Representative western blot of chromatin extracts incubated with α-CENP-A and α-H3 antibody. *Lower panel*: Quantification of SF-CENP-A and endogenous CENP-A signals. SF-CENP-A values were normalized to signals of endogenous CENP-A and are shown as mean ± SEM of three independent experiments. **C** Same as in **B** except that total soluble protein was analyzed. Statistical significance was determined by unpaired t-test with a significance threshold of $p<0.05$. ns, not significant, *$p<0.05$, ** $p<0.01$.

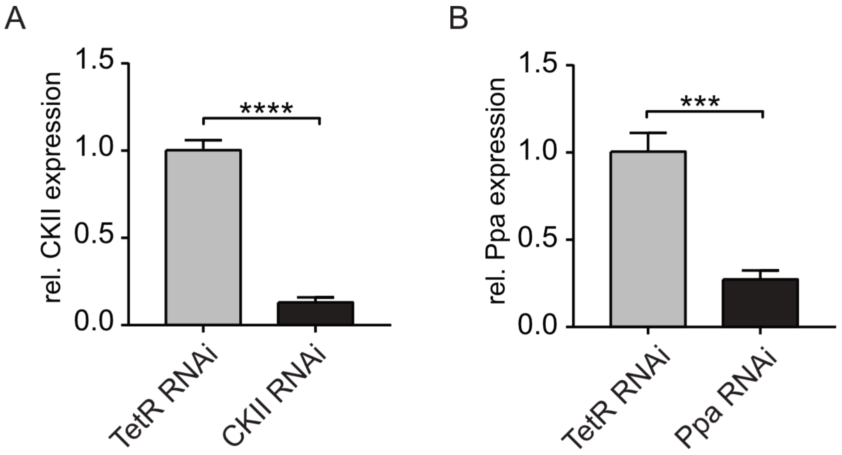

Supplementary Fig. S5. RT-qPCR of CKII (**A**) and Ppa (**B**) to determine RNAi efficiency in SF-CENP-A_wt cells used for CENP-A degradation assays. Gapdh was used as a reference. Means ± SEM relative to control-treated cells (TetR) of three independent experiments are shown. Statistical differences were determined by unpaired t-test. ***p<0.001, ****p<0.0001.

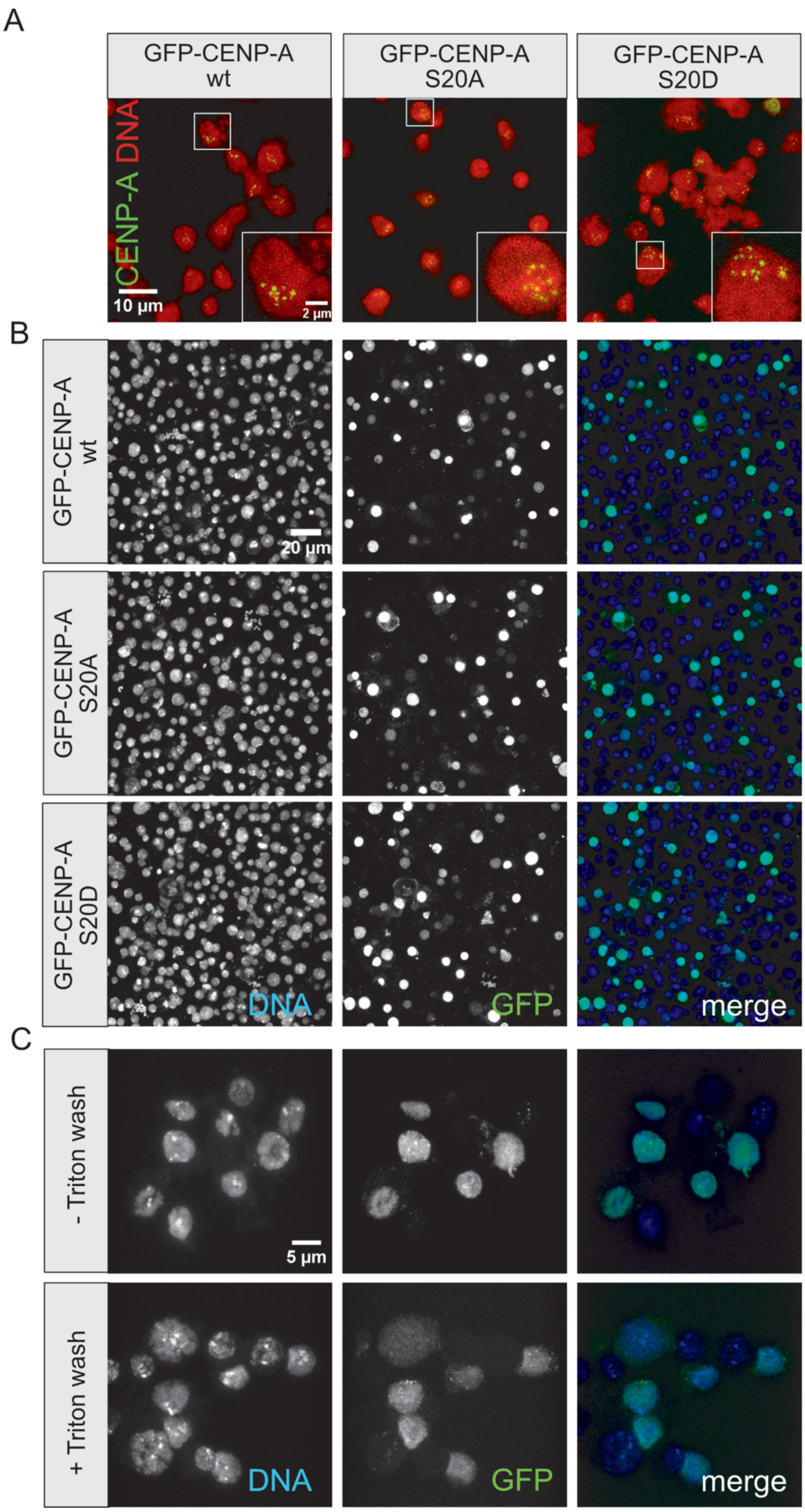

Supplementary Fig. S6. A Wild-type and S20 mutant GFP-CENP-A localizes to centromeric dots in the absence of $CuSO_4$ induction. Insets show 3x enlargement of the indicated nuclei. **B** Upon induction with $CuSO_4$ for 16 h, GFP-CENP-A proteins of the indicated cell lines show diffuse nuclear fluorescence. **C** GFP-CENP-A overexpressing cells were extracted by 3% Triton-X100 for 3 min prior to fixation and imaging to remove non-chromatin bound CENP-A. CENP-A was visualized by GFP fluorescence, DNA was stained with DAPI.

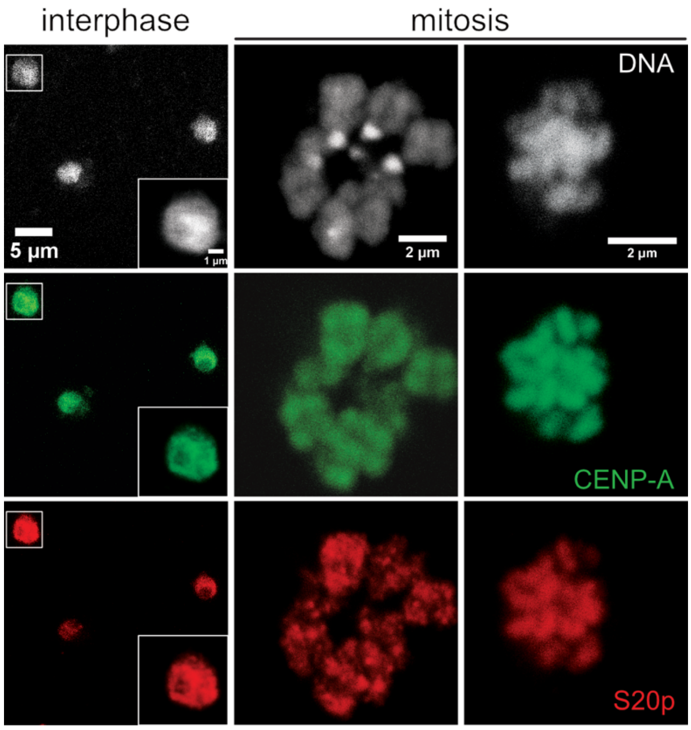

Supplementary Fig. S7. S20 phosphorylation on interphase and mitotic cells overexpressing CENP-A. *Left panels*, interphase cells. Insets are 2.5x enlargements of boxed nuclei. *Middle and right panels*, spreads of mitotic chromosomes of GFP-CENP-A_wt overexpressing cells. Cells were stained for GFP-CENP-A and phosphorylated S20 on CENP-A (α-S20p). Although CENP-A staining is diffuse throughout chromosomes, S20p staining can be detected in two forms, either diffuse (*right*) or in a dotted pattern (*middle*).

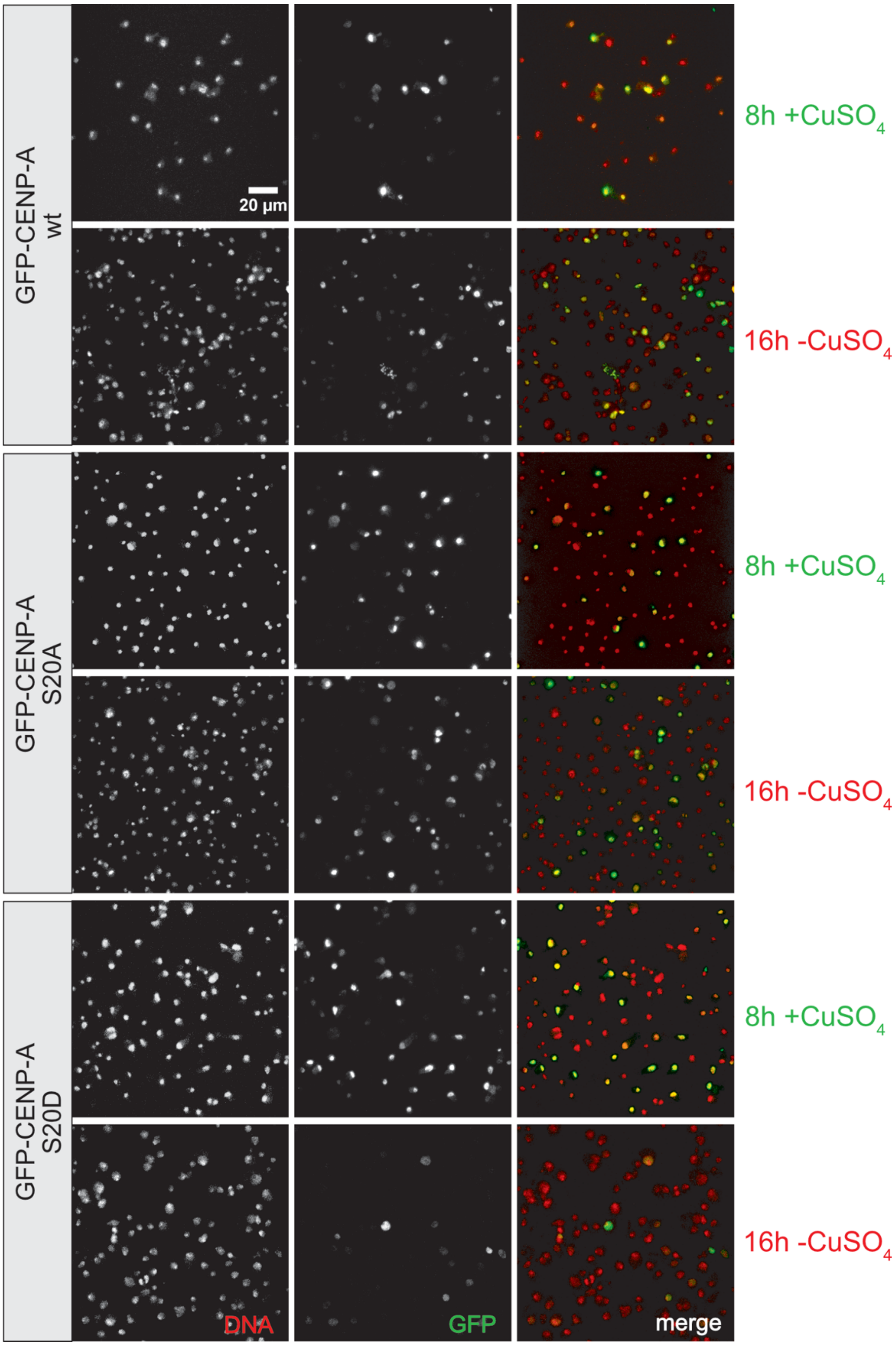

Supplementary Fig. S8. Indicated cell lines were induced to overexpress the respective CENP-A variant for 8 h before removal of $CuSO_4$ and chase for another 16 h. Cells were collected at both time points, stained for DNA and imaged using a confocal microscope. Maximum projections of z-stack images are shown. CENP-A was visualized by GFP fluorescence, DNA was stained with DAPI.

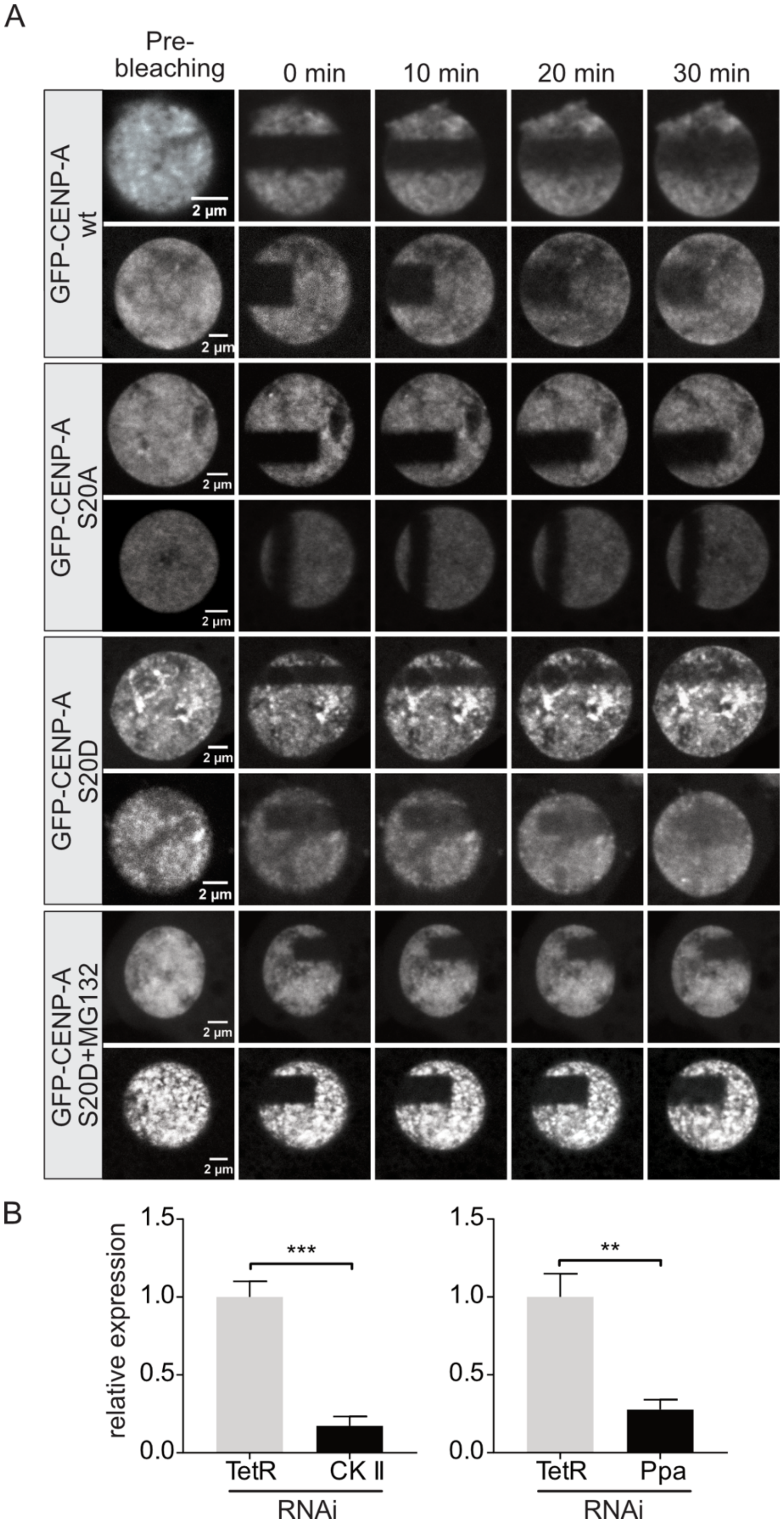

Supplementary Fig. S9. A Examples of nuclei quantified for FRAP analysis. Maximum projection of z-stack images of GFP-signals at the indicated time points are shown. **B** RT-qPCR of CKII and Ppa to determine RNAi efficiency in GFP-CENP-A_wt cells used for FRAP analyses. Gapdh was used as a reference. Means ± SEM relative to control-treated cells (TetR) of three technical replicates are shown. Statistical differences were determined by unpaired t-test. **p<0.01, ***p<0.001.

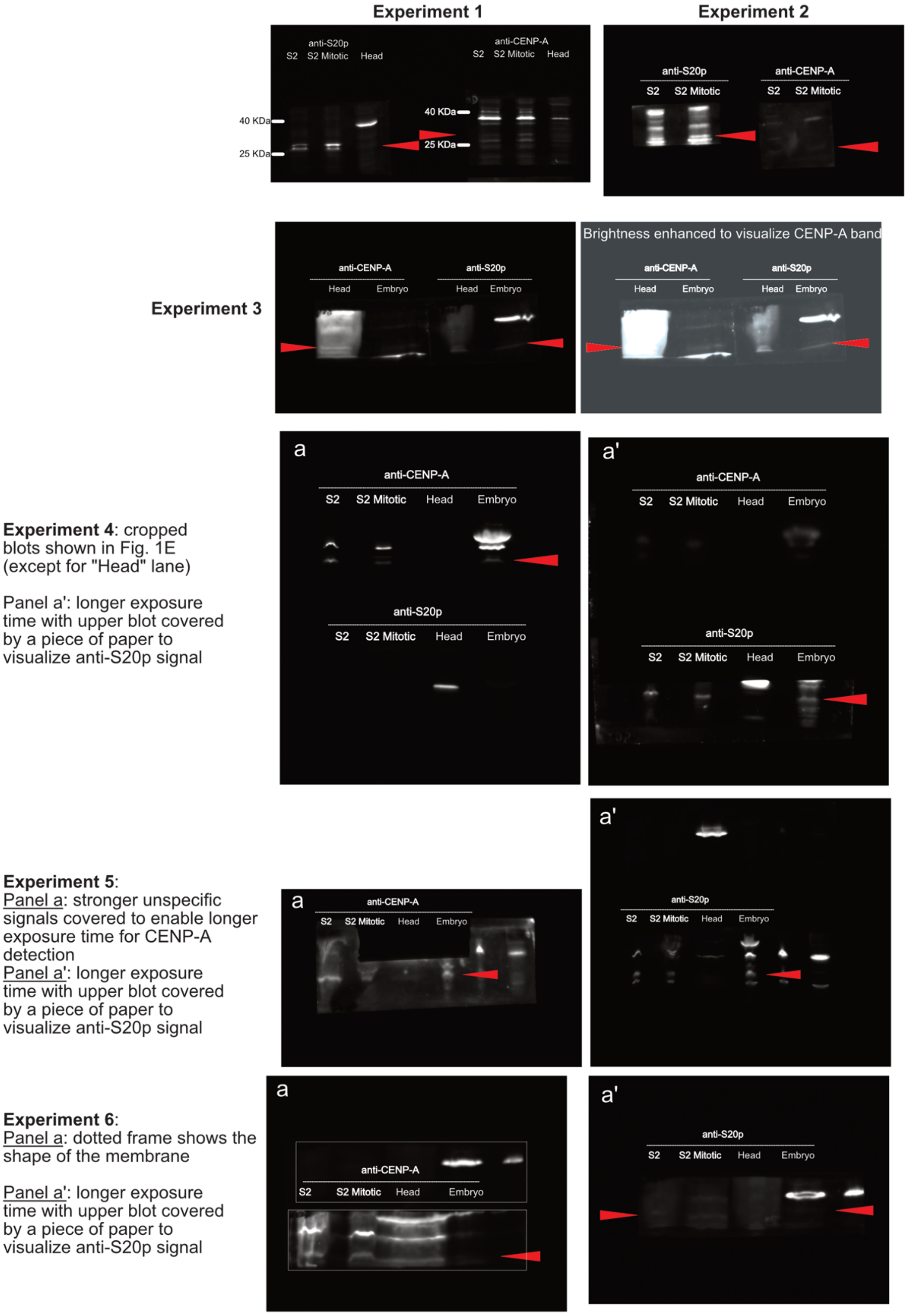

Supplementary Fig. S10. Raw western blot detection images of results from Fig. 1E. ECL signals were detected by a Fusion SL 3500 instrument (Vilber). Blots of 5 independent experiments with samples from S2 cells and mitotically arrested S2 cells (S2 Mitotic) and 4 independent experiments for embryo samples are shown. Samples were usually loaded in parallel onto two different gels for incubation with anti-CENP-A and anti-S20p antibodies,

respectively. For ECL signal detection, both blots were imaged together. Because the Fusion instrument restricts exposure time if there are strong signals, for detection of the weaker S20p signals, the second blot or parts of the same blot showing strong unspecific signals was covered with a piece of paper. Quantified bands are indicated by red arrowheads.

Experiment 1

Experiment 2

Experiment 3: cropped blots shown in Fig. 4C

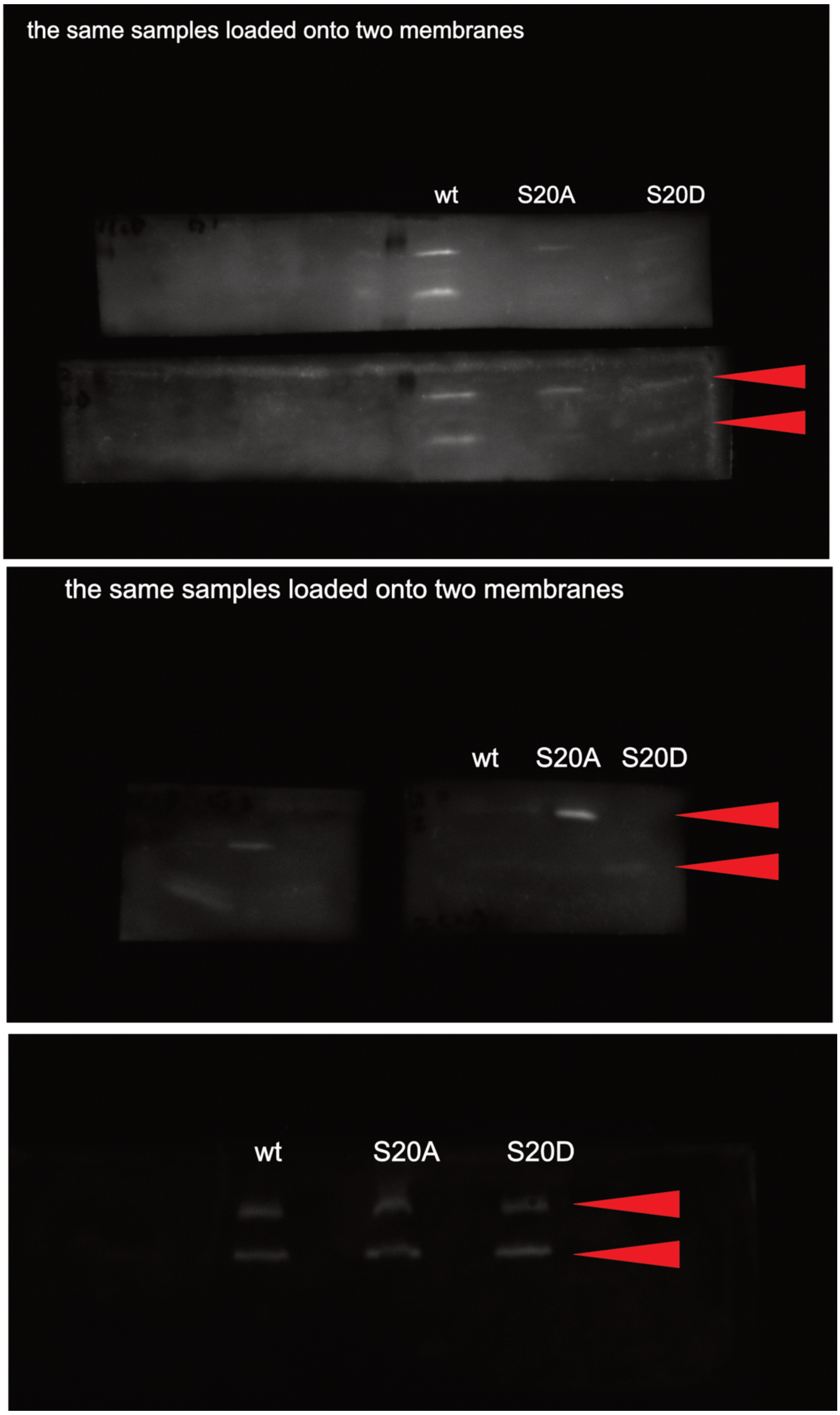

Supplementary Fig. S11. Raw western blot detection images of results from Fig. 4C. ECL signals were detected by a Fusion SL 3500 (Vilber) instrument. Blots of three independent experiments are shown. Different amounts of sample were loaded onto parallel blots, but only the one with the better signal was used for quantification (marked by red arrow heads).
